# Wheat-Based Glues in Conservation and Cultural Heritage: (Dis)solving the Proteome of Flour and Starch Pastes and Their Adhering Properties

**DOI:** 10.1021/acs.jproteome.3c00804

**Published:** 2024-04-04

**Authors:** Rocio Prisby, Alessandra Luchini, Lance A. Liotta, Caroline Solazzo

**Affiliations:** † Center for Applied Proteomics and Molecular Medicine, George Mason University, 10920 George Mason Circle, MSN 1A9, Manassas, Virginia 20110, United States; ‡ Independent Researcher for Museum Conservation Institute, Smithsonian Institution, 4210 Silver Hill Road, Suitland, Maryland 20746, United States

**Keywords:** wheat, starch, gluten, gliadin, leather, book conservation

## Abstract

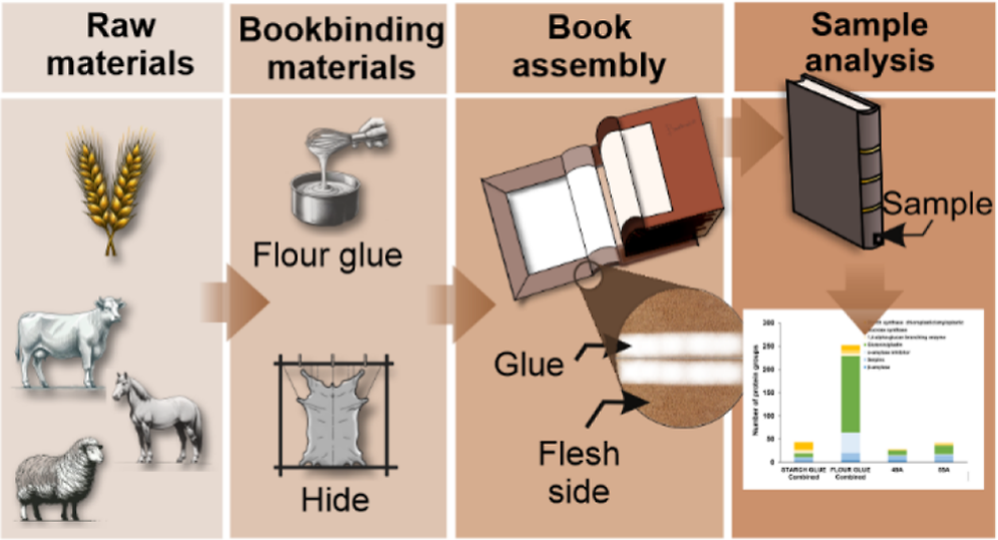

Plant-based adhesives, such as those made from wheat, have been prominently used for books and paper-based objects and are also used as conservation adhesives. Starch paste originates from starch granules, whereas flour paste encompasses the entire wheat endosperm proteome, offering strong adhesive properties due to gluten proteins. From a conservation perspective, understanding the precise nature of the adhesive is vital as the longevity, resilience, and reaction to environmental changes can differ substantially between starch- and flour-based pastes. We devised a proteomics method to discern the protein content of these pastes. Protocols involved extracting soluble proteins using 0.5 M NaCl and 30 mM Tris-HCl solutions and then targeting insoluble proteins, such as gliadins and glutenins, with a buffer containing 7 M urea, 2 M thiourea, 4% CHAPS, 40 mM Tris, and 75 mM DTT. Flour paste’s proteome is diverse (1942 proteins across 759 groups), contrasting with starch paste’s predominant starch-associated protein makeup (218 proteins in 58 groups). Transformation into pastes reduces proteomes’ complexity. Testing on historical bookbindings confirmed the use of flour-based glue, which is rich in gluten and serpins. High levels of deamidation were detected, particularly for glutamine residues, which can impact the solubility and stability of the glue over time. The mass spectrometry proteomics data have been deposited to the ProteomeXchange, Consortium (http://proteomecentral.proteomexchange.org) via the MassIVE partner repository with the data set identifier MSV000093372 (ftp://MSV000093372@massive.ucsd.edu).

## Introduction

### Historical Uses of Wheat Paste Adhesive and Application to Bookbinding and Paper Conservation

Wheat paste, crafted as a mixture of wheat flour or starch and water, was used in the past as an adhesive in arts and bookbinding. The adhesive properties of wheat paste come from gluten proteins, from which the term glue was derived. Flour is very sticky when wet and hardens upon drying. This effect is more exacerbated in wheat-flour-based glue than starch-based glue because of the gluten proteins.

Today, wheat starch paste is the most commonly used adhesive for repairs by book and paper conservators.^1,2^ Its uses for repair include hinging, mending, lining, facing, reinforcement, consolidation, or as a fixing media.^1^ Starch has also been used in leafcasting of parchment.^3^ However, little has been known about flour- and starch-based glues throughout history. The transition from using adhesives found in nature to production due to human manipulation has remained elusive.^4^ The earliest use of wheat paste might have occurred with the manufacture of papyrus by the Egyptians, as documented in A.D. 77 by Caius Plinius Secundus, Pliny the Elder, in his Natural History, Book VIII, which contains important details regarding the treatment Egyptians performed on wheat flour to extract the starch using boiling, straining, and diluted vinegar, and its application.^5^ Modern reconstruction of Egyptian papyrus has suggested that the flour paste was used to bind the constructed sheets of Papyrus (*Cyperus papyrus*, Cyperaceae) to each other to form the scroll.^6^ Microscopic analyses have identified wheat and barley grains at the junction of the sheets^7^ and revealed the presence of a starch layer (of unknown nature) in papyrus made up to 350 B.C.^8^

It is believed that wheat flour was used as a binding agent in Pompeian wall paintings based on the amino-acid composition of the binder,^9^ and wheat peptides have been identified in the mortar of ancient Chinese buildings.^10^ Shosoin documents and the book of Engishiki, from eighth century Japan, mention wheat and rice starch pastes as adhesives and ingredients of Mugi-urushi,^11^ a lacquer glue used to repair objects. While little is known about the use of wheat-based glue for historical artifacts and ornaments, the adhesive is known to have been used as a wallpaper paste, in the assembling and binding of books, mounting drawings or paintings, and, more generally, the binding of porous surfaces.^12^ Starch-based glues from wheat and rice have been used in East Asia in painting and calligraphy.^13^ Wheat starch grains were observed on paintings of Taoist priests, as flour glue was used to hold the painting to its support.^14^ Examination of Chinese paper by Julius von Wiesner (early 1900s) determined that starch was used as a sizing agent as early as A.D. 312,^15^ and more recently, proteomics analyses also identified wheat as a sizing agent in Tibetan papermaking (12–13th c.).^16^ In bookbinding, a flour-based paste is prepared by adding boiling water to the flour, forming a mixture that is used to attach the leather cover to the boards.^17^ This method has probably been used for centuries; often, the literature refers to “paste” for the binding agent of the leather cover to the board, but without more precision as to the nature of the paste,^18^ which can be assumed to be flour-based but could also include other types of gluing materials (animal glue, casein, egg, etc.).^19^

### Starch- or Flour-Based Glue

While the glycosidic components of starch (amylose and amylopectin) are well-known,^20^ the protein composition of both starch and flour glue has never been studied. Nowadays, starches are prepared by soaking the wheat flour repeatedly, kneading, and washing the formed stiff mass. Then, the mass is aged to allow separation of starch from gluten proteins and washed. Aging is a critical step during the separation; gluten coagulates and can be removed through screening, sieving, or centrifugation.^21,22^ Finally, starch is recovered through centrifugation and dried.^23−26^ In the past, starch was obtained by fermentation, a process that lasted for centuries; the grains were steeped in water to soften, then crushed and fermented, which destroyed the gluten, and the starch separated and dried.^27^ Unlike synthetic adhesives, starch-based paste is chemically stable, producing a durable and flexible bond,^28^ ideal for conservation purposes. Flexibility of restored areas is critical to avoiding further damage due to ruptures or splits.

Furthermore, starch-based paste does not cross-link over time, preserving solubility properties;^12^ the starch can be removed by swelling after moisture exposure.^20^ Starch resolubility is necessary for additional restoration steps; when aged, starch-based glues contract due to dehydration and fracture; therefore, they must be replaced to prevent further damage.^20^ Past conservation practices used glue to mount documents and textiles on solid supports. However, the flour-based glue can deteriorate for two main reasons: its own inherent aging process and its potential to serve as a food source for microorganisms. Both factors can damage objects, making removing the glue often necessary, usually through enzymatic methods.^20,29−31^ For these reasons, starch-based glues can indeed have the advantage of being less likely to interfere with analytical studies. Protein-based glue, such as flour glue, could introduce analytical ambiguities when identifying biological materials in an artifact, as the proteins from the glue may confound the identification of intrinsic biological material.

### Wheat Proteomics

The wheat seed is made of the embryo and the endosperm from which flour and starch are obtained.^27^ While flour contains the entire endosperm proteome, starch contains only a small portion of granule-associated and storage proteins.^25^ Previous studies have extensively examined the flour and starch proteomes due to their role in food allergies,^32,33^ dough properties,^34,35^ and significance in assessing grain yield and quality^36^ at different plant and grain development stages.

Nowadays, 2938 proteins have been identified in wheat’s endosperm.^37^ While these proteins differ structurally, they can be grouped based on their solubility in different solvents and buffers.^38^ Albumins are water-soluble proteins, while globulins are soluble in buffers containing salts such as sodium chloride (NaCl), representing 10–20% of endosperm proteome.^39^ Hydrophobic proteins, prolamines (gliadins), and glutenins are soluble in 70% ethanol and acidic solution containing a reducing agent, respectively.^34,39^ These groups represent 80% of the total protein in the flour and are equally represented.^34^

Despite the extensive literature on the wheat flour proteome, proteomics has not yet been explored to characterize wheat-based pastes in conservation. However, the starch extraction process from flour implies that flour- and starch-based pastes present different protein compositions, which can be used to identify unknown glues present in a sample. The recent identification by proteomics of wheat proteins in ancient paper and mortar^10^ highlights the potential of mass spectrometry-based analysis for wheat-based adhesives and the critical need to develop appropriate extraction protocols. Defining the proteomes of flour and starch pastes is the first step in identifying them in ancient samples and understanding how their differences contribute to both materials’ adhesive and aging properties. Here, we introduce a proteomics approach that sheds light on the protein content and profile of flour-based and starch-based pastes. A protocol was developed for the proteomics characterization of conservation grade glues and, as a proof of concept, was applied to a selection of historical bookbindings provided by the National Library of Medicine, where wheat paste was identified.

## Materials and Methods

### Wheat Paste Glue

The flour glue was prepared as follows: 30 g of commercial all-purpose flour was dissolved in 120 mL of Milli-Q water. The mixture was heated and stirred with a magnetic bar until it was boiled. Then, the mixture was removed from the hot plate and left at room temperature until it was cooled. The paste was stored in a refrigerator at 4 °C for future use.

### Starch Paste Glue

The starch glue was prepared following a method analogous to that employed by the Library of Congress:^1^ 30 gr of wheat starch from TALAS (no. 301) was dissolved in 150 mL of Milli-Q water. The mixture was heated and stirred with a magnetic bar until it boiled. Once translucent, the mixture was removed from the hot plate and left at room temperature until it was cooled. The paste was stored in a refrigerator at 4 °C for further use.

### Protein Extraction of Wheat and Starch Glue

Sequential extraction of proteins was performed: 100 mg of glue or raw material was mixed with 1000 μL of buffer 1 containing 0.5 M NaCl and 30 mM Tris-HCl and incubated at room temperature for 2 h in the rotator. Then, samples were centrifuged at 4 °C and 16,000 rpm for 15 min. The supernatant was extracted, desalted with PD-Spintrap G-25 (Cytiva) using 50 mM NH_4_HCO_3_, and saved for further proteomics analysis (fraction 1). The pellet was resuspended in 1000 μL of buffer 2 containing 7 M urea, 2 M thiourea, 4% 3-[(3-cholamidopropyl)dimethylammonio]-1-propa nesulfonate (CHAPS), 40 mM Tris, and 75 mM DTT. Extraction 2 was performed at 4 °C overnight on the shaker. Then, samples were centrifuged at 4 °C and 16,000 rpm for 15 min, and the supernatant was extracted. CHAPS was removed from the supernatant using Pierce Detergent Removal Spin Column, 0.5 mL (Thermo Scientific), following the manufacturer’s instructions. The supernatant was saved for further proteomics analysis (fraction 2).

### Protein Extraction of Leather Bookbinders and Control

A control sample of cattle vegetable-tanned leather V0 (top grain, natural color, Monseco Leather, Inc., Supporting Information Figure S1) was prepared by cutting 0.2 cm^2^ of leather, washing with two changes of Milli-Q water for 30 min each, and air-drying overnight. Three samples of vegetable-tanned bookbinding leather from a working collection of bookbindings at the National Library of Medicine were selected for this study (Supporting Information Figure S1):

- #46A is of unknown origin, a sample taken from the board: spine, near hinge, center. Full Leather. Color: raw umber, mocha. Year: unknown
- #49Ais of unknown origin, a sample taken from the board: Tail at spine edge (bottom board). Color: burnt sienna/tobacco. Year: 1871
- #55A is of unknown origin, a sample taken from the board: bottom left corner at tail center. Color: tan. Year: unknown

0.2 cm^2^ leather samples were submerged in 360 μL of buffer 1 containing 0.5 M NaCl and 30 mM Tris-HCl and incubated at room temperature for 2 h in the rotator. The supernatant (fraction 1) was extracted and desalted with PD-Spintrap G-25 (Cytiva) and 50 mM NH_4_HCO_3_, following the manufacturer’s instructions. Then, leather samples were briefly washed with 50 mM NH_4_HCO_3_, and extraction 2 was performed (fraction 2) with 200 μL of buffer 2 previously described. Extraction 2 was performed at 4 °C overnight on the shaker. Detergent removal was achieved using a Pierce Detergent Removal Spin Column, 0.5 mL (Thermo Scientific), following the manufacturer’s instructions. Leather samples were washed with three changes of 1000 μL of Milli-Q water for 20 min each. Milli-Q water was discarded, and extraction 3 was carried out using 110 μL of 50 mM NH_4_HCO_3_ at room temperature for 30 min in the rotator (fraction 3). Then, fractions 1, 2, and 3 were prepared for proteomics analysis.

### Sample Preparation for Proteomics Analysis

At room temperature, 150 μL of each fraction was reduced with 2 μL of 500 mM DTT for 30 min. Alkylation was followed by the addition of 4 μL of freshly prepared 500 mM iodoacetamide. Samples were incubated in the dark for 20 min. 2 μL of 0.5 μg/mL of Trypsin Gold, Mass Spectrometry grade (Promega) was added, and the solution was incubated at 37 °C overnight. The next day, 1.5 μL of TFA was added to stop the trypsin. Finally, the samples were cleaned using Pierce C18 Spin Columns following the manufacturer’s instructions with a minor modification for fractions 2 and 3: a first elution was performed using 20 μL of 30% acetonitrile; the column was placed in a new collection tube, and a second elution was performed, adding 20 μL of 70% acetonitrile to the resin bed. Centrifugation speed and duration remained the same. Tubes were stored at −20 °C.

### Mass Spectrometry Analysis

Dried samples were resuspended in 20 μL of formic acid, and 2 μL of the sample was analyzed with an Orbitrap Fusion TribridTM Mass Spectrometer (Thermo Scientific, Waltham, MA) coupled with a nanospray EASY-nLC 1200 UHPLC system. Reversed-phase chromatography separated the peptide mixture using PepMap RSLC 75 μm i.d. × 15 cm long with 2 μm, C18 resin LC (liquid chromatography) column (Thermo Fisher). 0.1% formic acid, and 0.1% formic acid and 80% acetonitrile were used. Sample peptides were eluted using a linear gradient of 5% mobile phase B to 50% mobile phase B in 90 min at 300 nL/min and then to 100% mobile phase B for an additional 2 min. The Thermo Orbitrap Fusion Tribrid Mass Spectrometer (Thermo Scientific) was operated in data-dependent mode in which each full MS (mass spectrometry) scan is followed by TopN MS/MS scans of the most abundant molecular ions with charge states from +2 to +4 dynamically selected for higher-energy C-trap dissociation (HCD) using a normalized collision energy of 35%. Two blanks were run between each wheat fraction sample to mitigate cross-contamination risks. For the leather samples, one blank was run between each sample.

### Bioinformatics Analysis

Mass spectrometry data were analyzed using PEAKS Studio PEAKS XPro.^40^ Starch and flour samples were searched against a Uniprot database of *Triticum aestivum* proteins (proteome entry UP000019116, downloaded and compiled on July 13th, 2023, from Uniprot.org(41)). Leather samples were searched against a compilated database of the *T. aestivum* proteome to which were added the Uniprot proteomes of *Bos taurus* (UP000009136), *Ovis aries* (UP000002356), and *Capra hircus* (UP000291000) downloaded September 28th, 2023 from Uniprot.org.^41^ Egg proteins were also added to the database from *Gallus gallus* (downloaded September 28th, 2023 from Uniprot.org(41)), and collagen sequences for *C. hircus* were downloaded on September 28th, 2023 from NCBI.^42^ The parameters used were the following: 5 ppm parent mass error tolerance, 0.25 Da fragment mass error tolerance, 1 max missed cleavage, and semispecific digest mode. Trypsin was selected as the enzyme for the starch/flour and Trypsin [D/P] for the bookbinders. Carbamidomethylation (C) was selected as a fixed modification, and the following variable modifications were chosen: oxidation (M), deamidation (N.Q.), and for the bookbinders, only hydroxylation (P). The number of maximum variable PTMs allowed per peptide was set at three (starch/flour) and six (bookbinders due to high levels of hydroxyproline in collagen). PEAKS PTM searches were performed to find further unspecified modifications, and data from PEAKS PTM were exported for further processing after filtering peptides using 1% FDR and proteins with −10 lg *P* ≥ 50.0 and a minimum of two peptides for protein validation.^43,44^ PEAKS PTM export files “DB search psm.csv” were used to calculate the deamidation of N and Q using the formula described in Mackie et al. 2018^45^ using the PSM’s area for intensity. A dedicated contaminant database was employed to identify and remove peptides originating from extraneous sources.^46^

## Results and Discussion

### Multistep Extractions

A proteomics workflow was established based on protein solubilities.^39,47,48^ Soluble proteins (electrically charged) were extracted in Buffer 1 using a salt solution (0.5 M NaCl and 30 mM Tris-HCl) used to primarily extract water-soluble (albumin) and salt-soluble (globulins) proteins. Tris-HCl was selected as a supplement to maintain the pH constant as the proteins elute. After centrifugation, the salt solution was extracted, and the pellet was resuspended in buffer 2, containing 7 M urea, 2 M thiourea, 4% CHAPS, 40 mM Tris, and 75 mM DTT. This step aimed at extracting insoluble storage proteins, including gliadins, glutenins, and other amphiphilic proteins. CHAPS was selected in this study for its ability to extract insoluble proteins from wheat. Both fractions were posteriorly analyzed by mass spectrometry. We found that a two-step extraction increased the protein recovery rate due to the diversity of biochemical identity in wheat proteins.

An additional extraction step was added for the leather samples with the aim of maximizing collagen extraction based on protocols used for archeological collagens using ammonium bicarbonate.^49^

### Starch Proteome

**Figure 1:**
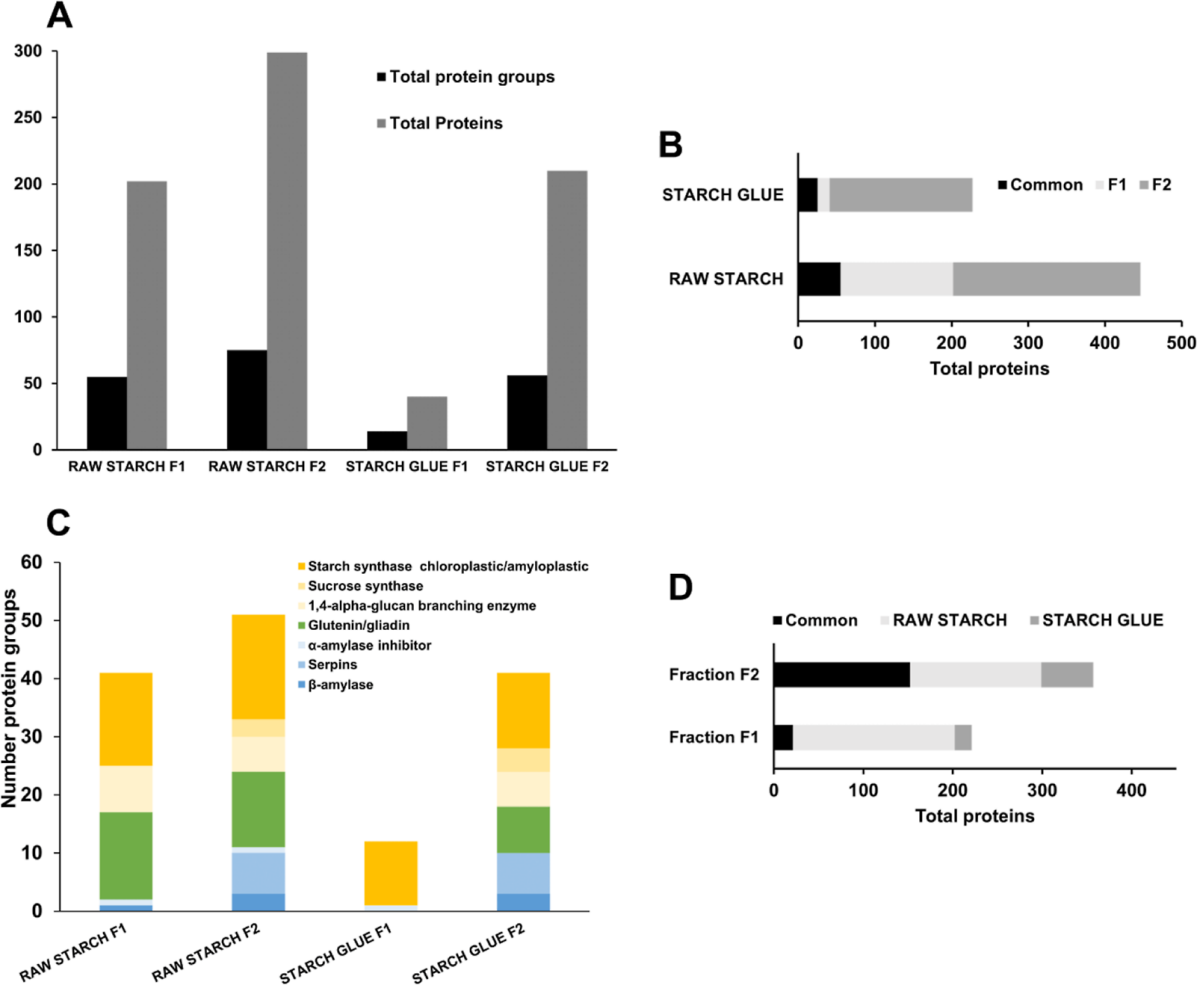
(A) Total number of protein groups and protein matches in fractions 1 and 2 of raw starch and starch glue. (B) Number of protein matches in fraction 1, fraction 2, and in both (common) in raw starch and starch glue. (C) Main protein groups identified in fractions 1 and 2 of raw starch and starch glue. (D) Number of protein matches in raw starch and starch glue and in both (common) from fraction 1 and fraction 2. Fraction 1 was performed using 0.5 M NaCl 30 mM Tris-HCl pH 8 buffer at room temperature for 2 h. Fraction 2 was performed using 7 M urea, 2 M thiourea, 4% CHAPS, 40 mM Tris, 75 mM DTT buffer overnight at 4 °C. Parameters are 1% FDR, protein score >50, minimum two peptides.

The two-step extraction was applied to raw and starch paste. Raw starch yielded a higher number of proteins than starch-based glue (Figure 1A,B). Fraction 2 showed a higher count of total protein groups and proteins in raw starch and starch-based glue when compared with fraction 1. This result agrees with the methodology employed in the starch preparation process, which involves extensive washing and sieving to isolate the starch granules. The observed reduction in water- and salt-soluble proteins is consistent with theoretical predictions. Due to the aqueous extraction conditions, the preferential removal of soluble, highly hydrophilic proteins can be expected, leaving behind predominately insoluble or starch-associated proteins in the resultant starch powder.

In raw starch, fractions 1 and 2 yielded a relatively balanced distribution of proteins, while the extraction in starch-based glue is highly skewed, with most of the proteins found in fraction 2 (Figure 1A,B). This unbalanced extraction could indicate changes in the mixture due to the glue preparation. When heated, noncovalent bonds holding the proteins to the starch granules can dissociate and promote reassociation with other proteins, changing the rhetorical properties of the mixture. Also, denatured and dissociated proteins may aggregate together, impeding extraction. Furthermore, when comparing raw starch to starch-based glue within each fraction, fraction 2 shows a higher overlap of proteins (152) than fraction 1, indicating a higher degree of homogeneity (Figure 1D). In contrast, most proteins identified in fraction 1 are found only in raw starch (Figure 1D).

Starch synthase proteins were the starch-granule-associated proteins found in the highest numbers across all fractions (Figure 1C). 1,4-α-Glucan branching enzymes were found in both soluble and insoluble forms of raw starch, but only the insoluble form of starch glue, while sucrose synthase is insoluble in both raw starch and glue. These proteins are firmly adjoined within the starch granules.^50^ Thus, buffer 2 is demonstrated to be more efficient in disrupting starch associations and potential protein aggregates.

Raw starch contained significantly more counts of glutenin and gliadin than starch-based glue. Like other starch-associated proteins, the molecular interactions among glutenin, gliadins, and starch may be affected by the heat, stirring, and dehydration required in the glue preparation. Heat causes gliadins and glutenins to polymerize^51^ due to increased interchain linkages via disulfide bonds.^52^ This association between glutenins and gliadins is highly sensitive to both the duration and the temperature of the heating process involved in preparing the glue. As a result, these proteins are expected to be isolated more easily from the raw starch than from the glue sample.

### Flour Proteome

**Figure 2:**
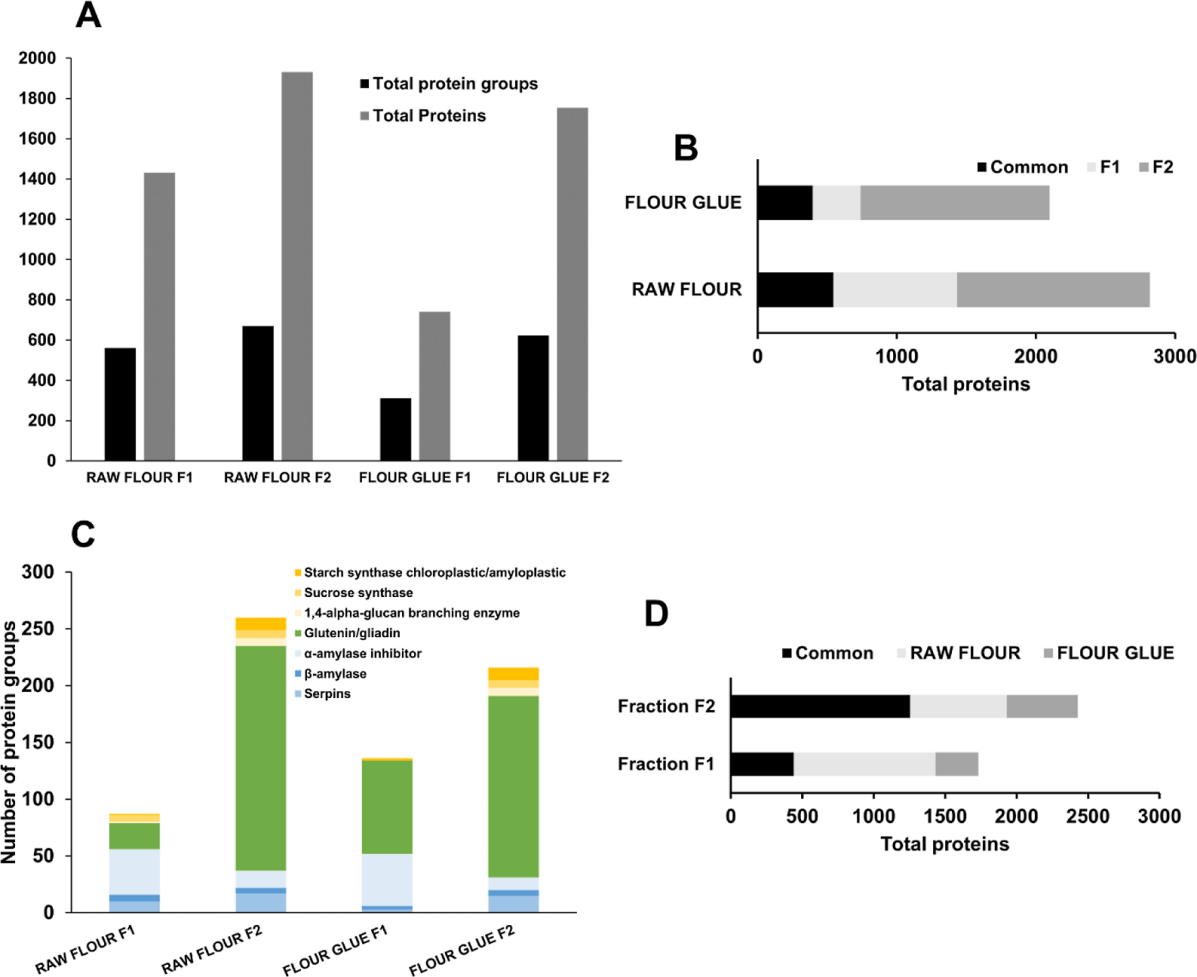
(A) Total number of protein groups and protein matches identified in fractions 1 and 2 of raw flour and flour glue. (B) Number of protein matches in fraction 1, fraction 2, and in both (common) in raw flour and flour glue. (C) Main protein groups identified in fractions 1 and 2 of raw flour and flour glue. (D) Number of protein matches in raw flour and flour glue, and in both (common) from fractions 1 and fraction 2. Fraction 1 was performed using 0.5 M NaCl 30 mM Tris-HCl pH 8 buffer at room temperature for 2 h. Fraction 2 was performed using 7 M urea, 2 M thiourea, 4% CHAPS, 40 mM Tris, and 75 mM DTT buffer, overnight at 4 °C. Parameters are 1% FDR, protein score >50, minimum two peptides.

The in-house-prepared flour paste proteome was compared to the proteome of raw flour. Figure 2A shows the numbers of protein groups and protein matches in fractions 1 and 2 of the raw- and flour-based glue. In fraction 1 (water- and salt-soluble proteins), the number of identified proteins and protein groups increased 2-fold in the raw flour paste compared to the flour glue, a trend similar to the one observed with starch, where a markedly higher number of unique proteins were found in the raw material rather than the glue (Figures 1B and 2B). Indeed, nearly 60% of proteins extracted in fraction 1 are found only in raw flour, likely becoming insoluble in the paste form (Figure 2D). Reduced solubility in the flour paste due to denaturation or structural modifications could force proteins to elute in fraction 2 rather than fraction 1.

As with starch, Figure 2B shows that most proteins extracted from each fraction are distinct (about 19% are found in both fractions), but more proteins are extracted in fraction 2 (more than 50% of unique proteins in both raw flour and paste), indicating either the presence of more intrinsically insoluble proteins or the transition of some initially soluble proteins into insoluble ones. Proteins found in raw flour eluting in fraction 1 may elute in fraction 2 after the flour is processed into glue, while other proteins may never elute due to the tight interaction with the starch increased by heat, leading to an overall decrease in total identifications (Figure 2A).

Raw flour analysis exhibited greater proteomics complexity than flour-based glue; this proteomic diversity indicates a broader range of proteins with different biological and structural functions within the material (Supporting Information_Wheat.xlsx). Similar to starch-based glue, heating, and stirring can cause alterations in protein structure and molecular interactions, reflected by the differential elution, thus reducing the sample’s proteomics diversity and detectability. Despite these changes, a significant number of proteins were conserved among the samples, indicating an inherent robustness to heat and mechanical stress or functional dispensability. On the other hand, the relative concentration of certain specific protein groups (e.g., glutenin/gliadin and others) slightly differed between raw and flour-based glue. This suggests again that the glue preparation process may selectively affect specific protein categories, favoring the elution of the unchanged one.

The predominant protein categories delineated in Figure 1C are shown in Figure 2C, corresponding to the various flour fractions. Gluten proteins (glutenins/gliadins) manifested in substantial concentrations, despite fraction 1 having a reduced count of protein groups and being designed to extract exclusively soluble proteins. It is known that certain members of the gliadin group are soluble in salted water. The buffer in this experiment contained 0.5 M NaCl, which might have catalyzed a structural change in the more hydrophilic gliadins, facilitating their solvation and consequent elution. α-Amylase inhibitors, an example of a constituent of the water-soluble albumin group, were detected in fraction 1.

Within fraction 1, there is a particularly large increase in the number of gliadin/glutenin protein matches in the paste state. Denaturing and unfolding caused by elevated temperatures can break intermolecular interactions, promoting a more extended configuration, which might increase their solubility in aqueous solutions or buffers.^53^ Gliadins are more soluble than glutenins; therefore, more gliadins are expected to elute in fraction 1 in raw flour and glue; glutenins may elute less efficiently in fraction 1. Furthermore, glutenin/gliadins’ presence in both fractions, but predominantly in fraction 2 for flour and glue, suggests their inherent solubility and presence in both free and starch-bound states. The significant increase of these proteins in fraction 2 indicates that raw flour has a high proportion of these proteins in a more hydrophobic form due to their intrinsic properties or association with other flour components.

Proteomic diversity between raw and flour-based glue sheds light on each sample’s composition, structural diversity, and functionalities potentially lost when transformed into a derivative product like glue.

### Comparing Starch and Flour Proteomes

**Figure 3:**
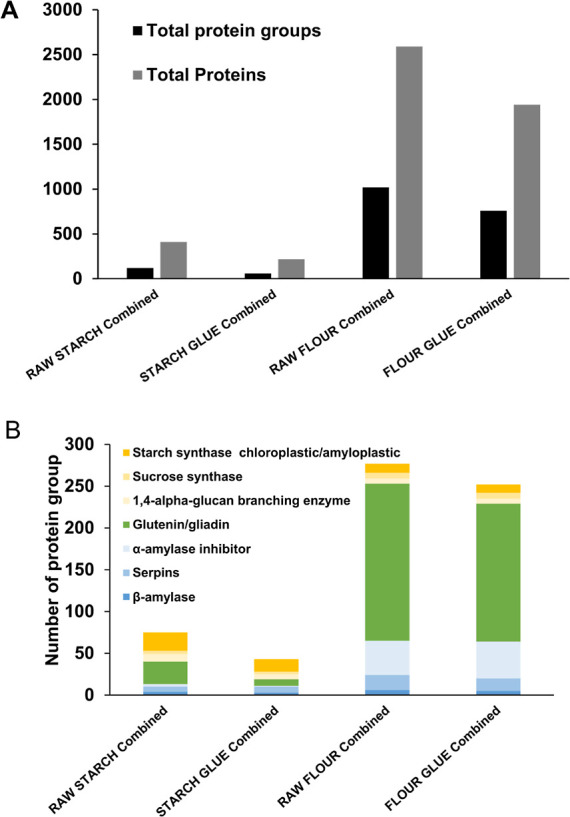
(A) The bar graph shows the abundance of the total number of protein groups (black) and total proteins (gray) when fractions 1 and 2 are combined across four samples: raw starch, starch glue, raw flour, and flour glue. Notably, the raw flour combined sample exhibits the highest number of total proteins. In contrast, starch glue combined samples show the lowest counts in both metrics, proteins, and protein groups. (B) Profiling of specific protein groups across the combined samples. The stacked bar graph shows the number of protein groups in the same combined samples.

To assess the proteome of each material, in raw and paste forms, fractions 1 and 2 were combined in a unique peaks search. For both starch and flour, a loss of proteins occurs when the materials are in their paste form compared to the raw form, as was observed in each individual fraction (Figure 3A). The presence of 759 protein groups in the flour-based glue compared to 58 in the starch-based glue highlights the richer proteomic profile of the former. This difference is expected due to the nature of the starch isolation process to produce the starch. In addition to a higher proteomic complexity, flour-based glue showed a relatively higher percentage of proteins in both fractions than starch-based glue. This suggests that some proteins in the flour-based glue might have a “balanced” affinity for both extraction environments. Starch- and flour-based glues showed a higher percentage of proteins specific to fraction 2 than raw starch and flour. This suggests that in both cases, many proteins are more hydrophobic or firmly bound to the matrix and thus extracted in the second fraction.

Raw flour shows the highest presence of most protein groups (Figure 3B), especially marked by the significant amounts of glutenin/gliadin. On the other hand, the starch paste has notably limited amounts of glutenin/gliadin and α-amylase but a higher number of starch synthase proteins. Proteins in this group are known to be granule-bound synthases responsible for the elongation of glucan chains of amylose and amylopectin; therefore, it is not surprising that they were abundantly present. On the other hand, its abundance in flour-based paste was lower (Figure 3B), as well as its protein coverage (Supporting Information_Wheat.xlsx); this could have resulted from poor extraction or concealing due to a higher abundance of other proteins.^54^ When preparing pastes, the granules undergo significant swelling upon being combined with water and subjected to heat, thus dehydration. This event prompts a structural alteration in the proteins proximal to the granules, predominantly due to protein denaturation, which subsequently impacts their elution during extraction. Starch synthase’s role in determining the adhesive’s viscosity and elasticity remains ambiguous; however, it may function as a distinctive indicator for material characterization in conservation endeavors.

Gluten proteins comprising soluble gliadins and insoluble glutenins were the predominant protein category in the flour glue. While their presence was notably diminished, they were still detectable in starch-based glue. In an ideal extraction scenario, starch should be free from gluten components.^55^ However, the presence of gluten, as evidenced by our analysis, indicates that the extraction process is not fully efficient. Several contributors could be considered: (a) gluten and starch may be intimately intertwined at the molecular level. A high amount of gluten is removed during extraction, but residual attached gluten might persist. (b) Gluten proteins might have some affinity to starch, leading to a more persistent bound. Even though the extraction process targets this dissociation, the sheer diversity in gluten’s proteinaceous composition can mean that some protein subfractions have a higher affinity to starch. (c) Alongside, affinity can also be influenced by the quality of the raw material and the grain age, making the extraction process less optimal.^56,57^

Besides gluten proteins, α-amylase inhibitors (αAI) were the most abundant proteins in flour and had the best protein coverage (Supporting Information_Wheat.xlsx). αAI are proteins that inhibit the enzymatic activity of α-amylases. These inhibitors are classified as members of the albumin family, encompassing a wide range of water-soluble proteins in wheat seeds. The antimicrobial activity of αAIs plays a significant role in plant defense against pathogens, such as fungi and bacteria. Plus, their antimicrobial activity may be associated with other members of the same group; disruption of microbial cell membranes is needed to interfere with microbial metabolic pathways.^58,59^

While most of the proteins in the albumin group are lost, αAI could be more tightly attached to the granule structure due to the abundance of cysteine residues and disulfide bonds. When starch granules are hydrated, protein solvation causes conformational rearrangements; proteins expose hydrophobic regions while disulfide bonds are buried, forming a “coat” on the starch granule. When dehydrated during glue preparation, starch gelatinizes, water is transferred from the proteins to the starch, and proteins form a stiff network around the starch, preserving αAI throughout the starch preparation process.^60^ In conservation, the antimicrobial activity of α-amylase inhibitors could be critical for book preservation and restoration.^31,61^

Percentage coverages in the gluten group were not as high as those in the amylase and some other proteins (Supporting Information_Wheat.xlsx). A high composition of glutamine and proline characterizes this group of proteins.^62^ This abundance can influence the protein’s structure, making it more compact and less accessible to proteolytic enzymes. Gluten proteins have fewer charged amino acid residues compared with other proteins. This low overall charge can influence how the protein interacts with the solution, further affecting its solubility and, by extension, its detection. Gluten proteins’ solubility is influenced by their size and arrangement; large proteins and polymeric arrangements are less soluble. Also, trypsin cleaves proteins at specific sites, the C-termini of lysine (K) and arginine (R). When proteins lack these amino acids, trypsin digestion may be less efficient, leading to fewer detectable peptides and, thus, lower percentage coverages in mass spectrometry.^63^

On the other hand, although comprising 2% of the gluten proteins, cysteine (C) amino acids may significantly influence protein behavior. Cysteine amino acids can form intra- and intermolecular interactions via disulfide bonds, stabilizing protein complexes. Disulfide bonds are susceptible to thermal disruption and dehydration, leading to protein conformation and solubility changes.^64^

While “gluten” is broadly employed to encompass glutenins and gliadins, these proteins exhibit distinct characteristics, imparting flour-based glues with different properties. Upon rehydration of flour to prepare the glue, glutenins take up water, bestowing the adhesive with elasticity and strength due to the comprehensive matrix of monomers encircling the starch granules. In contrast, gliadins provide the adhesive with viscosity due to the system’s less coherent organization of gliadin monomers, which acts as a glutenin plasticizer. Owing to these dynamics, both protein groups are critical in producing an adhesive with a smooth texture and thin consistency when wet and a robust and reliable bond once dried.

Starch glue owes its adhesive properties to molecular interactions and modifications during glue preparation.^65^ Starch is primarily made of two types of polysaccharides–amylose and amylopectin. Amylose is composed of d-glucose units linked through α-(1–4) glycosidic bonds, while amylopectin is highly branched through α-(1–6) links, and α-(1–4) glycosidic bonds linking d-glucose units.^66^ The glucose units’ abundant hydroxyl (–OH) groups allow extensive inter- and intrahydrogen bonding. This bonding capability leads to a cohesive network that imparts adhesive properties. When applied to a surface, van der Waals forces and mechanical interlocking of the starch molecules with the microscopic irregularities of the surface improves adhesion. Van der Waals forces are responsible for bonding smooth surfaces, while mechanical interlocking is common in paper and other porous surfaces where the glue can penetrate more easily.^67^

When the starch glue is prepared, the starch powder underwent rehydration and heating. During this heating, the starch granules experience gelatinization. In this phase, water molecules penetrate the semicrystalline structure of the starch granules, causing them to swell. As the temperature continues to rise, these granules eventually burst, releasing amylose and amylopectin.^68^ Amylose is more susceptible to this process due to its intrinsic linearity compared to amylopectin. During retrogradation, amylose and, to a lesser extent, amylopectin align and form hydrogen bonds, leading to crystallization or reassociation.^69^ This results in the formation of a structured gel matrix. This gel matrix imparts specific properties to the starch-based glue; it provides firmness and enhances its binding strength, making it an ideal adhesive. Furthermore, the semicrystalline structure provides enhanced stability and resistance to breakdown over time.

Building on the details mentioned above about the behavior of starch during gelatinization and retrogradation, the presence, role, and influence on adhesive properties of intrinsic proteins in the starch matrix emerge as a potential area of investigation. Incorporating external protein sources into the starch mixture enhances adhesive properties.^70^ However, it has been demonstrated that due to its hydrophobic properties, gluten’s contribution to adhesion is much lower compared to starch.^71^ This suggests intrinsic proteins may behave differently, enhancing or harping adhesive properties; proteins can inhibit or promote starch retrogradation, contingent upon exposed residues. The gelatinization process can be affected by three main interactions: charged residues can engage in charge–dipole interactions with phosphate groups bound to starch, hydrophobic residues impede amylose release and reassociation, and hydrophilic residues promote water and molecular suppleness.

Another layer of complexity is introduced when interchain disulfide bonds are formed. Such bonds could amplify the effects of retrogradation and increase the robustness of the adhesive. Contrarily, glycosidic bonds between starch and proteins, formed at high temperatures, could restrain retrogradation.^72^ Therefore, understanding the role of proteins in the properties of starch-based glue holds significant potential for tailoring the glue for different purposes.

### Protein Identification in Historical Bookbindings

The methodology developed for characterizing wheat proteins in bookbinding leathers was employed on three samples of vegetable-tanned leather from the National Library of Medicine, which were previously analyzed at the Smithsonian’s Museum Conservation Institute (unpublished 2019 study). We selected samples 49A and 55A, which contained wheat traces, and 46A, which did not, along with a control sample of vegetable-tanned leather from cattle. Analyzed samples were approximately 0.2 cm^2^ in size, sourced from a research collection of leather covers detached from their original books, where the small sample size was deemed inconsequential to the project’s objectives. Instead, the focus was on maximizing wheat protein recovery, a critical aspect given the glue layer’s minimal thickness and potential lack of uniformity compared to the leather. Future research will endeavor to define an analytical threshold that considers factors such as sample accessibility, the pursuit of minimally destructive sampling techniques, degradation, and the resolution of the instruments used.

**Figure 4:**
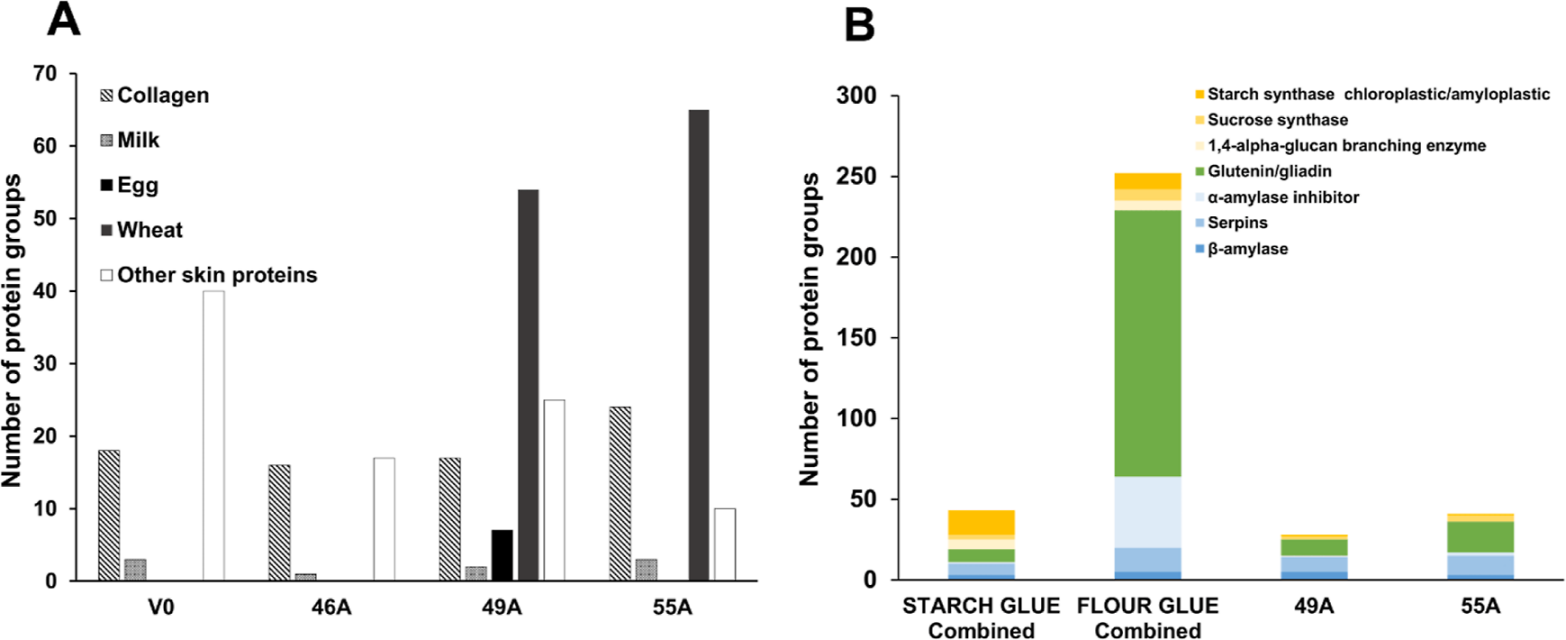
(A) Categories of proteins identified in the leather samples V0, 46A, 49A, and 55A. Among the samples, 49A and 55A show a high wheat protein concentration. (B) Main wheat proteins identified in the leather samples compared to the starch and flour pastes.

The 3-step extraction applied here was meant to improve the identification of the wheat proteins compared to the previous one-step extraction and propose a protocol suitable for wheat proteins and collagen from the leather and other adhesive/additive proteins. The three fractions were combined for PEAKS and searched against a database containing collagen for cattle, sheep, goat, milk, and chicken egg proteins and wheat proteins from *T. aestivum*, based on the protein identification from the previously analyzed samples. The different categories of protein identified (keratins excluded) are listed in Figure 4A.

Species identification of the binding leather was based on collagen identification, especially collagen type I α 1, collagen type I α 2, and collagen type III α 1, the main collagen chains found in skins (Supporting Information_Leathers.xlsx andSupporting Information Tables S1 to S4). All binding samples were identified as Bovidae and were consistent with the species identification from the unpublished data: cattle for V0, likely goat for 46A, sheep for 49A, and cattle for 55A. As expected from the previous unpublished analysis, wheat proteins were not found in the control and leather samples 46A but were found in binding samples 49A and 55A and are presumed to be from the paste used to glue the leather to the board. In contrast, cattle collagen was found in 46A, suggesting that animal glue rather than wheat paste was used for bookbinding. While collagen identification is equivalent for goat and cattle in that sample, the identification of the leather as goat and the glue as cattle is more likely since cattle is more commonly used in animal glue.^73,74^ Furthermore, the animal glue can be in part or completely attributed to hide glue due to the presence of collagen type III α 1, characteristic of hide glue.^73^ In the analysis of binding sample 49A, egg white proteins, exhibiting up to 74% protein coverage for chicken ovalbumin, were detected, suggesting their application in the book’s finishing processes. Specifically, the utilization of egg white as an adhesive agent for edge gilding^74^—a decorative technique evident in 49A (Figure S1)—underscores its role in historical bookbinding practices. Conversely, chicken egg proteins were absent in sample 46A. However, a targeted ornithological proteomic analysis revealed the presence of three bird ovalbumin peptides, one of which can be uniquely attributed to duck species. This nuanced detection of duck ovalbumin, also corroborated by a previously unpublished study, will be further explored for its implications in a forthcoming publication.

Milk was found in all samples and could be part of a casein-based glue or, more likely, was applied during the leather treatment to add a soft finish to the skin.^74,75^ In some samples, a low number of peptides were identified. The fact that the books were once used and manipulated outside the laboratory raises contamination concerns, as milk is a common food product. The details of the milk protein identification are shown in Supporting Information with protein and peptide identification given in Tables S5–S9 for all samples, including starch glue. Surprisingly, the starch glue contained a substantial number of milk peptides, which we attribute to the material itself (likely added during manufacturing of the starch powder, intentionally or not; see Supporting Information_TEXT). The potential source and function of egg, milk, and wheat as binding or finishing materials warrant future studies leveraging the methods developed herein.

**Figure 5:**
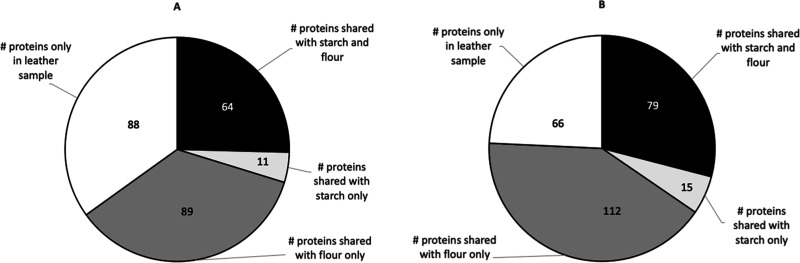
Number of protein matches identified that are shared with starch or flour pastes in (A) binder 49A and (B) binder 55A.

The breakdown of the main proteins found in wheat, compared to starch and flour glues, is shown in Figure 4B for the two binding samples, 49A and 55A. The binding samples show a more significant proportion of gliadin/glutenin proteins than starch synthase proteins, and the serpin proteins are present in numbers similar to those of the flour paste. The relative proportions of these different proteins show more consistency with flour paste than with starch paste. Figure 5 shows the number of protein matches identified by their accession numbers present in binding samples 49A (Figure 5A) and 55A (Figure 5B) and shared with starch and/or flour. In both cases, the highest numbers of proteins identified are also found in the flour paste rather than the starch paste. Finally, Table 1 shows all protein families identified in the binding samples and their correspondence in the starch and flour pastes. Besides tubulins, the proteins found in the binding samples and flour but not starch are all enzymes (e.g., ATP synthase subunit β, dehydroascorbate reductase, peroxidase, etc.).

**Table 1:**
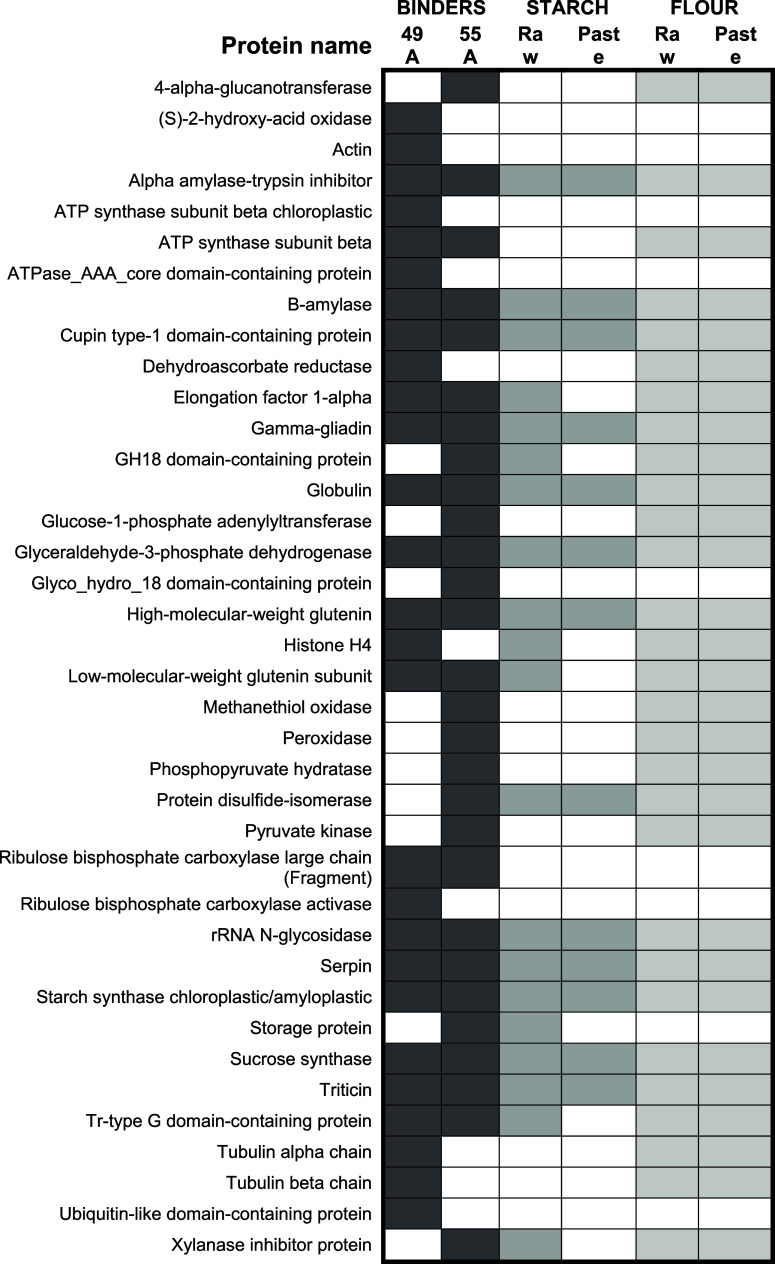
Wheat Proteins Identified in the Binding Samples

The proteins identified with the best percentage coverages were β-amylase and serpins, perhaps unsurprisingly, since these proteins are more amenable to trypsin digestion due to regular R and K, in contrast with gliadins and glutenins.

The gliadin family has three types: α, γ, and ω gliadins, of which only α and γ contain cysteine residues. Both α and γ were found in the starch and flour. In the binding samples, however, only the γ type was found. γ-Gliadins, characterized by their abundance of disulfide bridges, possess distinct molecular stability.^76^ γ-Gliadins form four intramolecular disulfide bonds against three for the α-gliadins. These bonds provide structural integrity and stability to proteins under denaturing conditions like heat. Proteins rich in disulfide linkages are more resistant to proteolytic enzymes, reducing degradation in environments where proteases are active.^77^ In addition, there is more β-sheet structure in the γ-gliadins (20–23%) than in the α-gliadins (11–12%),^78^ which could provide additional chemical resistance. In the context of bookbinding samples or adhesives, the inherent stability of γ-gliadins may allow them to maintain their adhesive properties over time, resisting degradation that could compromise the integrity of the adhesive bond. Further, the abundance of disulfide bridges may enable cross-linking with starch molecules and other matrix components, leading to a more intricate and stable adhesive. This unique molecular architecture could explain their exclusive presence in the binders compared with their α and omega counterparts.

### Fractions in Bookbinding Samples

Table 2 shows the distribution of proteins and protein groups by fractions in the two binding samples, where wheat was identified. In both cases, the wheat proteins were mainly extracted in fraction 2, followed by fraction 3. Fraction 1, aimed at extracting water and salt-soluble proteins, extracted a few proteins in the binding samples: β-amylase (both samples) and low-molecular-weight glutenin and cupin type-1 domain-containing protein (in 55A). One of the significant proteins identified in fraction 1 of the flour samples (but not starch), α-amylase inhibitor (an albumin protein), was not identified in fraction 1 of the binding samples and was present in a small proportion in fraction 2. This protein and other proteins found in fraction 1 in flour might be more susceptible to degradation due to their solubility or lack of binding properties. While fraction 1 did not contribute many unique protein matches (Table 2), it slightly improved the protein coverage of some of the proteins identified, for example, β-amylase in binding sample 49A (Table 3). However, more unique protein matches were found in fraction 3 (Table 2 and Supporting Information_Leathers.xlsx). In binding sample 55A in particular, some proteins that could be key to flour rather than starch identification were identified only in fraction 3, i.e., 4-α-glucanotransferase, glucose-1-phosphate adenylyltransferase, and phosphopyruvate hydratase, pyruvate kinase, showing that there is some value in adding a third extraction step.

**Table 2:**
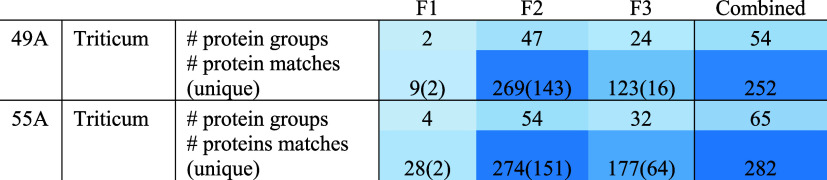
Number of Protein Groups and Protein Matches Identified in the Binding Samples by Fraction

**Table 3:**
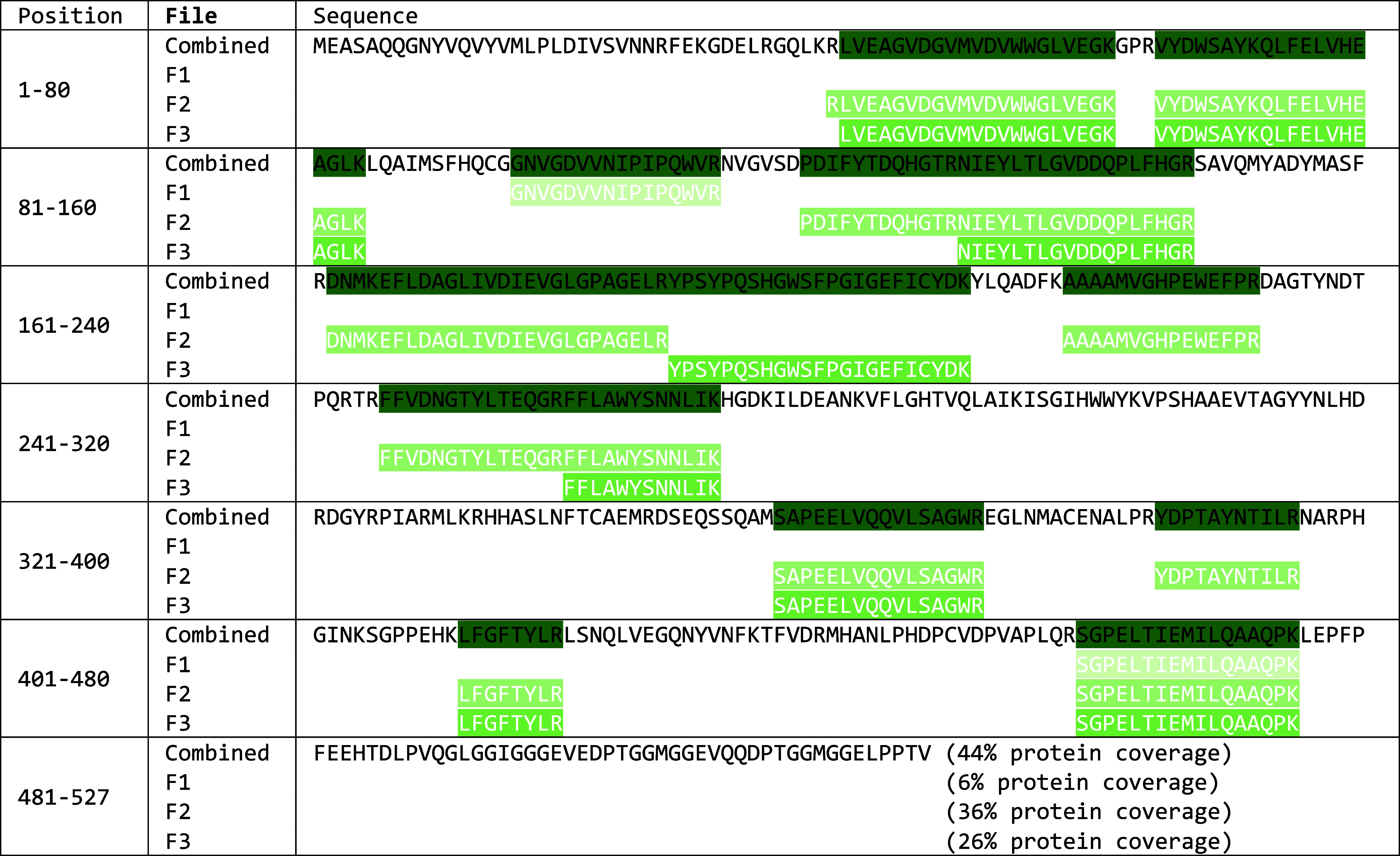
Sequence Identification in >tr|A0A3B6U9P0|A0A3B6U9P0_WHEAT Β-Amylase OS = *T. aestivum* OX = 4565 PE = 3 SV = 1^a^

a The parts of the sequence identified in each fraction F1, F2, and F3 and in the combined search are highlighted in different shades of green and the respective percentage protein coverage given after the sequence.

### Deamidation in Bookbinding Samples

Deamidation of asparagine (Asn) and glutamine (Gln) was calculated as a measure of the preservation of the leather and the adhesive. Gluten proteins are particularly rich in glutamine, containing 30–35% of the residue.^79^ Deamidation of glutamine in gluten, the main binding proteins of flour glue, could lead to changes in the secondary and tertiary structures of gliadin and glutenin and increase the solubility and flexibility of the adhesive, which in historical samples could contribute to weakening and loss of the adhesive.

**Figure 6:**
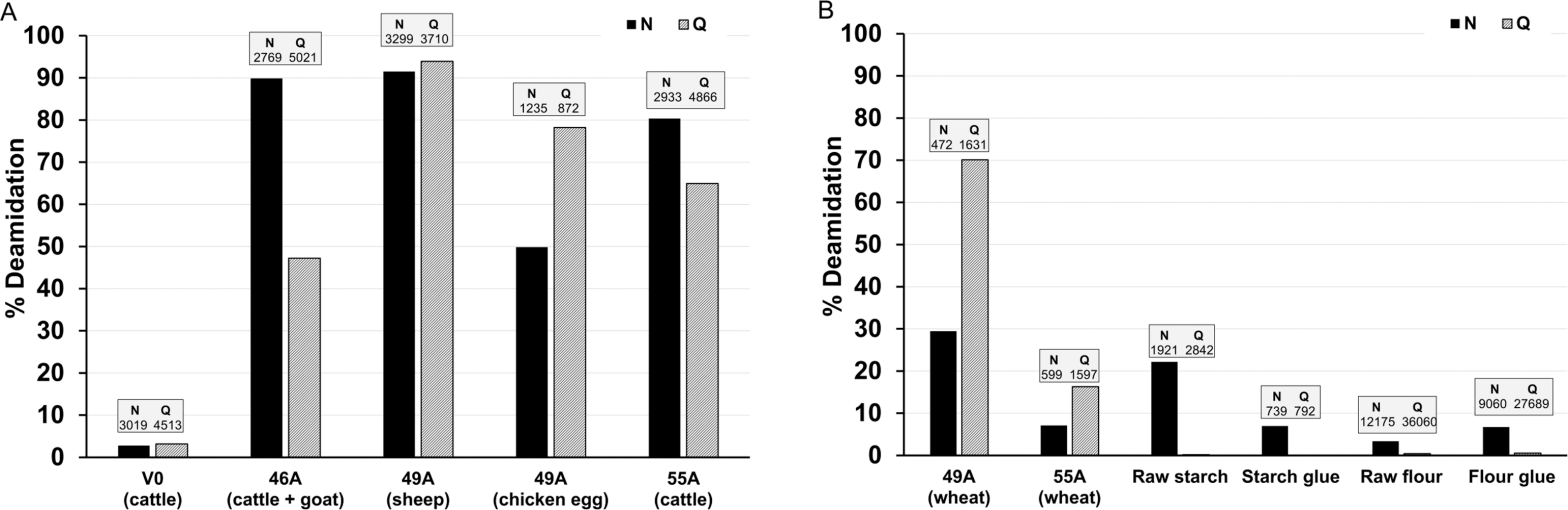
Percentage of deamidation calculated for Asn N and Gln Q in (A) leather and egg proteins and (B) wheat proteins. The total number of N (asparagine) and Q (glutamine) residues on which the calculation was based is indicated above each bar. Milk peptides are not included due to the low number of peptides containing N and Q for calculation.

The percentage of deamidated Asn and Gln residues was calculated separately for each category of proteins identified with a significant number of N and Q residues: skin and egg proteins (Figure 6A) and wheat proteins (Figure 6B) (see Methods). The leather deamidation was expectedly lower for the control sample of vegetable-tanned leather, which was kept in a stable environment (room temperature and dark) but was significantly higher for the binders.

Asparagine, which has a rapid rate of deamidation,^80,81^ was found to be highly deamidated in all binders’ leather (above 80%). In binder 46A, collagen deamidation calculated for either goat or cattle species had similar levels (data not shown), resulting from the high level of sequence homology between the species. The overall deamidation (combining both species) shows lower levels of glutamine deamidation than in 55A and 49A. Deamidation was particularly high in binder 49A, dated from 1871, a possibly older binder than 46A and 55A. However, research on bone collagen has shown that deamidation does not necessarily correlate with age but is instead dependent on environmental conditions.^80^ While little research has been done to understand the effect of ancient tanning methods on collagen deamidation, it has been shown that deamidation of parchment and other skin objects is more dependent on the production techniques and history of the objects than age.^82,83^ Processes such as liming (dehairing), deliming, and tanning are done under alkali and acidic conditions. Vegetable-tanned leathers have a low pH, which could accelerate deamidation with time.

**Figure 7:**
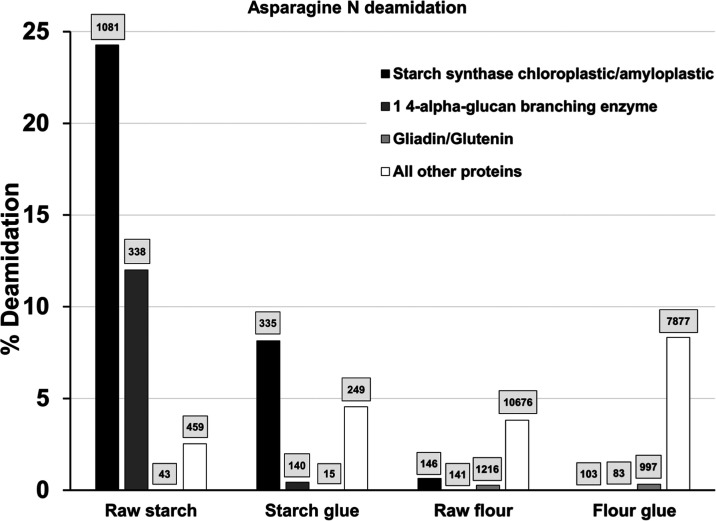
Asparagine deamidation in wheat samples. Percentage of deamidation calculated for Asn N in key protein families. The total number of N (asparagine) residues on which the calculation was based is indicated above each bar.

Wheat deamidation (Figure 6B) indicates that deamidation for asparagine, which has a theoretically faster rate of deamidation than glutamine,^84^ had occurred in the starch and flour materials, while glutamine deamidation was negligible. Asparagine deamidation was higher in starch, despite the higher abundance of Asn residues in the flour samples, which might be a result of the extraction process of starch from flour. While deamidation increases from raw flour to flour glue, which is expected due to the use of heat and water,^85^ the opposite occurred from raw starch to starch glue. Interestingly, when breaking down the overall percentage of deamidation by proteins or a family of proteins (Figure 7), asparagine deamidation is mainly supported in starch by the starch synthase chloroplastic/amyloplastic and 1,4-α-glucan branching enzyme proteins; proteins that are directly involved in starch biosynthesis. The decrease in overall deamidation is due to the decrease of deamidation in these proteins. A decrease in the deamidation of starch synthase chloroplastic/amyloplastic in glue compared to raw is also observed in flour. However, this protein’s contribution in Asn residues to the total number of Asn residues is negligible.

As mentioned above, starch granules undergo significant swelling in the presence of water and heat, leading to gelatinization;^86^ the crystalline regions within the granules are broken and collapse.^87^ As the paste cools, amylose and amylopectin reassociate into an ordered structure in a process called retrogradation.^72^ Starch-bound proteins have been shown to inhibit starch retrogradation^88^ through alterations in the granule’s surface chemistry, affecting the dynamics of starch–water interactions. These intrinsic components also serve as sites for the attachment of exogenous proteins and enzymes, potentially modulating the retrogradation pathway. Additionally, the formation of covalent disulfide bridges between protein molecules facilitates starch retrogradation, while glycosidic linkages that develop between starch and proteins under high-temperature processing conditions are likely to inhibit retrogradation.^72^ The decrease in deamidation of starch-bound proteins is significant and implies that the changes in conformation from raw material to glue have an impact on the extraction of deamidated peptides, possibly due to changes in the association between glycosidic components of the starch granules and starch-bound proteins.

In flour, the contribution to deamidation is mainly supported by the category falling under all other proteins (mainly albumin/globulin proteins). In both starch and flour, deamidation increases in this category of proteins. In the gluten (gliadin/glutenin) fraction with very few Asn residues, little or no deamidation is observed in either starch or flour.

**Figure 8:**
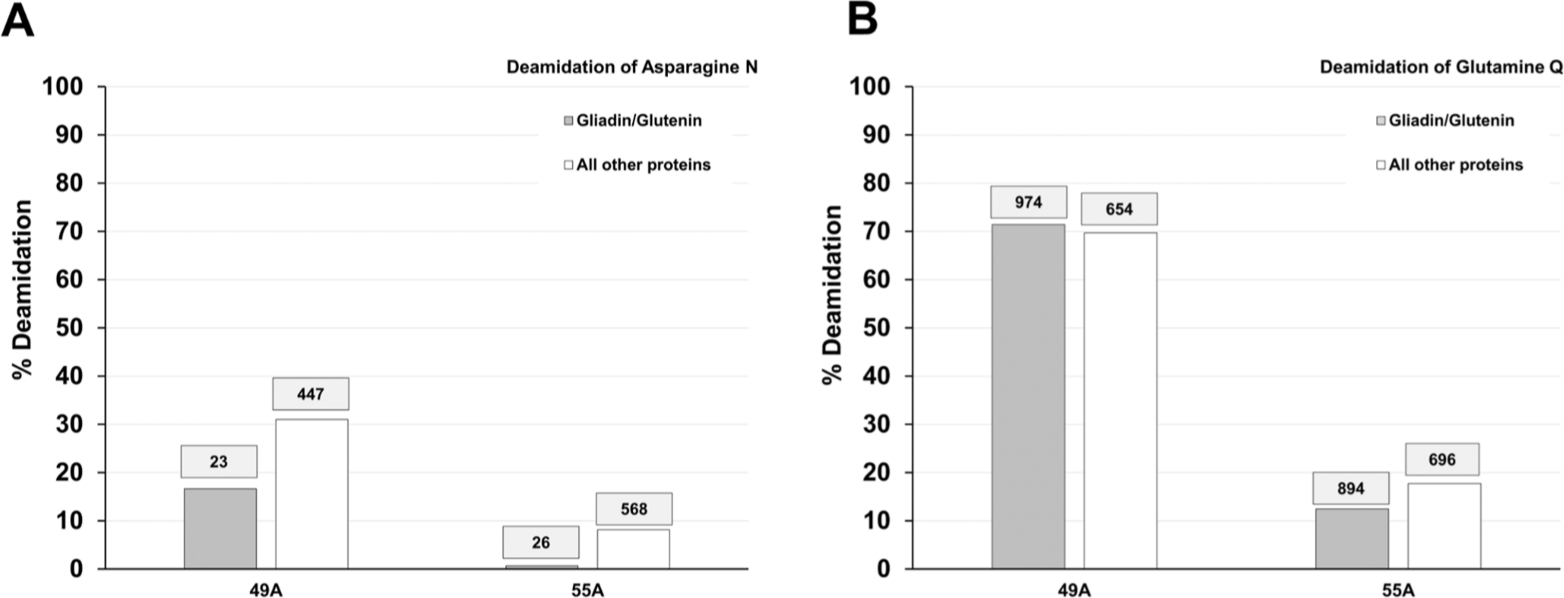
Asparagine and glutamine deamidation of wheat proteins in the bookbinding samples. The percentage of deamidation calculated for Asn N and Gln Q is shown for gluten and nongluten proteins. The total number of N and Q residues on which the calculation was based is indicated above each bar.

Glutamine deamidation was equally high in all historical bookbinding leather but was measured in the skin proteins (Figure 6A) at an expectedly lower levels than asparagine in samples 46A and 55A. Surprisingly, glutamine’s deamidation measured in wheat proteins (Figure 6B) was higher than asparagine’s deamidation in both binders 49A and 55A. Deamidation of Asn and Gln is detailed in Figure 8 for gluten and nongluten proteins. Since starch-bound proteins were identified in the bookbinding samples with no or less than 10 sites for deamidation, the calculated percentages of deamidation were not deemed representative and are not included in Figure 8. Deamidation of asparagine in the bookbinding samples (Figure 8A) was consistent with deamidation observed in the flour glue sample; i.e., it was higher in the nongluten proteins than the gluten proteins (which contributed, however, a small number of Asn residues). The levels of deamidation in 55A for Asn are similar to the ones observed in the flour glue. In spite of the presence of less Asn residues in 49A, the levels of deamidation are higher than in 55A and consistent with the deamidation observed in leather proteins. This indicates that the lower levels of deamidation of asparagine compared to glutamine are unlikely to result from a loss of Asn residues. The levels of deamidation of glutamine shown Figure 8B indicate a similar contribution to deamidation from gluten and nongluten proteins.

The abundance of glutamine in wheat has been estimated at about 10x that of asparagine (19.3 to 35.4 g per 100 g protein for Gln, compared to 2.0 to 3.9 g per 100 g protein for Asn in wheat protein ingredients^89^). The higher abundance of glutamine in wheat proteins increases the probability of deamidation events simply due to a greater number of glutamine residues. However, the percentage of observed deamidation is not a direct function of residue abundance; rather, it indicates the rate at which these residues undergo deamidation under the experimental conditions. Gln and Asn’s deamidation rates are subject to the influence of several factors. These include the immediate protein environment surrounding the residues, the side chain accessibility, and the tertiary and quaternary structures within which the residues are situated.^90^ Our findings indicate that Gln exhibits a higher deamidation percentage on a per-residue basis than Asn. These results suggest a kinetic preference for the deamidation of Gln in the protein structures examined in this study. The proteins’ three-dimensional conformations may position Gln residues in close proximity to structural motifs that accelerate deamidation or increase exposure to solvents and catalytic groups within the proteins.

Furthermore, introducing additional negative charges through deamidation may lead to protein unfolding, potentially exposing more residues to deamidation.^91^ It has also been posited that Gln undergoes more rapid deamidation than Asp when located at the N-terminus.^92^ Although the role of Gln deamidation as a molecular clock is well-recognized,^93^ kinetic studies focusing on Gln are less prevalent in the literature than those on Asn.^94^ Nonetheless, recent investigations have started to reveal unpredicted trends toward increased rates of Gln deamidation compared to Asn.^95,96^ To fully understand what factors impact deamidation in starch and flour glues, future studies should examine the glues as a simple system and conduct aging experiments on the glues alone; the biochemical mechanisms underpinning our observations involve a complex interplay of structural, thermal, and kinetic factors, which merit detailed scrutiny in future studies.

In the context of the bookbinding samples, the differences in deamidation rates between Gln and Asn might also have been influenced by the stability of the protein matrix in the leather. Since both collagen and wheat have high levels of glutamine, deamidation in one system could influence deamidation in the other. Indeed, when comparing samples 49A and 55A to 46A, glutamine deamidation is proportionally higher in both binders with flour glue compared to asparagine deamidation. Glutamine deamidation is even slightly higher than asparagine’s in the leather proteins for 49A (Figure 6A). Deamidation of both Gln and Asn is likely to be influenced by the tanning agent in leather;^97^ the conformational stability of the collagen triple helix and its interaction with the tanning agents could differentially protect or expose these residues to conditions conducive to deamidation. Finally, the independent calculation of the deamidation of the egg proteins in 49A, for which a high number of peptides were identified, follows the same trend as that for the skin and wheat proteins, with a significantly higher level of deamidation for Gln than Asn.

Incorporating additional proteinaceous binders such as ovum, casein, or additional collagenic substances in bookbinding may substantially modulate the deamidation kinetics of leather-bound proteins. These ancillary proteins, each with unique amino acid profiles and deamidation tendencies, engage in intricate biochemical interactions with both leather and adhesive matrices. Such interactions could conceivably alter the microenvironment and structural configuration of glutamine and asparagine residues, potentially rendering them more susceptible to deamidation processes.^98^ Including these heterogeneous protein systems could thus synergistically affect deamidation dynamics, further influencing bookbinders’ degradation and conservation characteristics. Therefore, this study suggests that the different protein systems (leather, glue, and finishes) are likely to interact with each other and perhaps provoke accelerated rates of deamidation, which in turn might impact the materials’ aging properties.

## Conclusions

This study describes for the first time the proteome of wheat starch-based and flour-based pastes, highlighting the higher protein diversity of the flour-based paste, while starch-based paste is mainly composed of starch granules-associated proteins. The difference in composition was used to characterize the wheat-based adhesive used in two bookbindings. A multistep protocol was developed to first extract water-soluble (albumins) and salt-soluble (globulins) proteins, followed by the extraction of insoluble storage proteins, including gliadins, glutenins, and other amphiphilic proteins. The study also shows the decreasing complexity of the proteomes once starch and flour are made into pastes.

Wheat flour has been used since ancient times due to its viscoelastic properties. These properties are conferred by glutenin and gliadin proteins accumulated in grains during its development. In the past, the quality and type of the flour would also have been different, resulting in variable quality and durability of the pastes. For instance, refined white flour, as the ones used in the experiments, only became commonplace after the roller milling process appeared around 1870, which eliminated all bran and germ residues from the flour.^99^ Stone-ground flour was coarser and contained a variable amount of grain coating and germ. Some of the proteins identified in the binding samples but not in the flour samples could result from the less-refined flour used in the historical glue.

Wheat varieties were more numerous in the past; bread wheat landraces have been replaced by monocultures of pure genotypes, resulting in a loss of genetic diversity.^100^ It was found that the high-yielding modern varieties have a lower protein content and decreased proportions of gliadins but an increase in glutenins.^101,102^ The gliadin/glutenin proportion similarly varies by wheat species: while glutenin is the dominant fraction in common wheat, gliadin makes up the highest fraction in species of so-called ancient wheats (einkorn, emmer, and spelt) as well as having an overall higher protein content.^103,104^ Environmental conditions such as soil, temperature, and irrigation also play a role in protein composition.^104^ In parallel to a decrease in overall protein content in modern bread wheat, the starch content has increased.^105^ In the past, a pure starch paste might, therefore, have been challenging to achieve, based on a lower starch content and imperfect techniques to separate starch from flour, resulting in a starch paste containing a higher protein content. Our analyses here show that, even in commercial starch, a significant amount of proteins, including some gluten, is found. Finally, in addition to genetic and environmental factors that likely influenced the gluten and starch content of ancient wheat, the process of extracting starch from flour, as well as the many recipes to prepare starch and flour paste, must have resulted in a range of adhesives with various viscosities and strength,^1^ all of which could be reflected in the proteome of the final product. As seen in the bookbinders, the application and aging of the materials result in the loss of more soluble proteins.

Understanding the chemical changes of wheat-based adhesives, such as deamidation, which could contribute to a more soluble glue, is essential to adopting best practices for restoration and conservation purposes. Further research on wheat pastes and other plant adhesives should consider the wide range of parameters, such as preparation recipes, additives, and aging, as well as the wide variety and composition of the raw material.

## Data Availability

The mass spectrometry proteomics data have been deposited to the ProteomeXchange, Consortium (http://proteomecentral.proteomexchange.org) via the MassIVE partner repository with the data set identifier MSV000093372 (ftp://MSV000093372@massive.ucsd.edu).

## Supporting Information Available

All proteins identified in the wheat samples (XLSX)
All proteins identified in the leather samples (XLSX)
Photographs of the leather control and bookbinding samples; peptide markers for species identification of cattle, goat, and sheep in control samples V0, bookbinding samples 46A, 49A, and 55A; milk-derived peptide identification in starch; and milk-derived peptide identification in leather samples (PDF)
