# Supplementary material for: Wheat-Based Glues in Conservation and Cultural Heritage: (Dis)solving the Proteome of Flour and Starch Pastes and Their Adhering Properties
Source: J Proteome Res. 2024 Apr 4;23(5):1649–65. doi: 10.1021/acs.jproteome.3c00804 (PMC11077587; doi:10.1021/acs.jproteome.3c00804)
Supplement: Supplementary file 3 — pr3c00804_si_003.pdf [file pr3c00804_si_003.pdf]

SUPPORTING INFORMATION_TEXT

Wheat-based glues in conservation and cultural heritage: (dis)solving the proteome of flour and starch pastes and their adhering properties

Rocio Prisby[1*], Alessandra Luchini[1], Lance A. Liotta[1], Caroline Solazzo[2]

[1]Center for Applied Proteomics and Molecular Medicine, George Mason University, 10920 George Mason Circle, MSN 1A9, Manassas, VA 20110
[2] Independent researcher for Museum Conservation Institute, Smithsonian Institution, 4210 Silver Hill Road, Suitland MD 20746, USA

Table of Contents

Name	Description
**Bookbinding samples, p2**	**Photos of bookbinding samples and sampling location**
Figure S1, p2	Leather control and bookbinding samples
**Species identification in the bookbinding leather samples, p3**	**Bioinformatics analysis and species identification in bookbinding samples**
Table S1, p3-4	Species markers in bookbinder sample V0
Table S2, p5	Species markers in bookbinder sample 46A
Table S3, p6	Species markers in bookbinder sample 49A
Table S4, p7	Species markers in bookbinder sample 55A
**Identification of milk proteins, p8**	
Tables S5a and S5b, p9-10	Protein and peptide identification in Starch glue
Tables S6a and S6b, p11	Protein and peptide identification in V0
Tables S7a and S7b, p11-12	Protein and peptide identification in 46A
Tables S8a and S8b, p12	Protein and peptide identification in 49A
Tables S9a and S9b, p12-13	Protein and peptide identification in 55A
**Datasets**	**Proteins identified in all samples**
Supporting Information_Wheat.xlsx	Proteins identified in raw starch, starch glue, raw flour, and flour glue in fractions 1, 2, and combined
Supporting Information_Leathers.xlsx	Proteins identified in the combined runs for all the leather samples (V0, 46A, 49A, and 55A), and for samples 49A and 55A, all proteins identified in fractions 1, 2, and 3.

Bookbinding samples

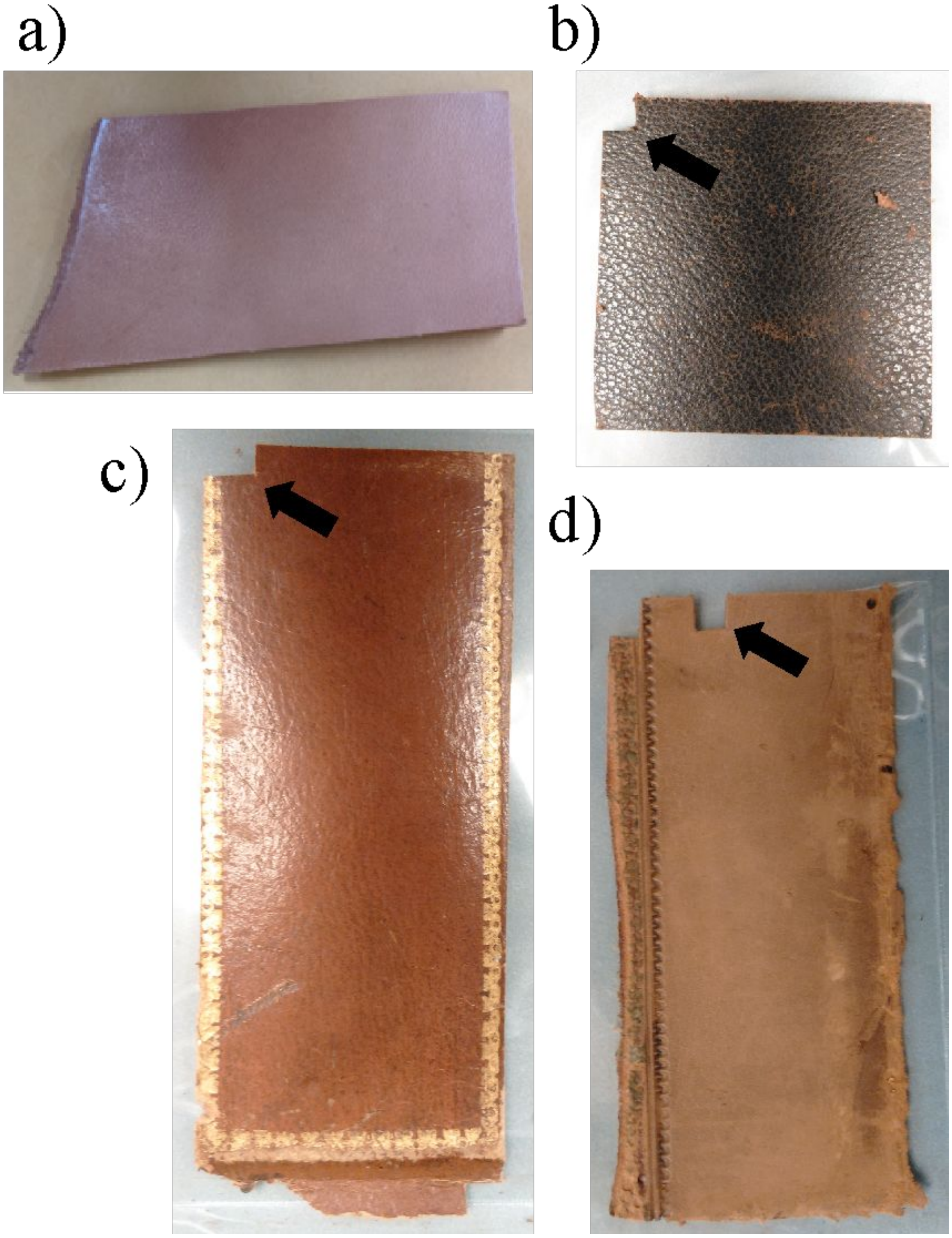

Figure S1. Leather control and bookbinding samples: a) V0, cattle vegetable-tanned leather (top grain, natural color, Monseco Leather, Inc.); b) bookbinding sample #46A is of unknown origin, a sample taken from the board: spine, near hinge, center. Full Leather. Color: raw umber, mocha. Year: unknown; c) bookbinding sample #49A is of unknown origin; a sample taken from the board: Tail at spine edge (bottom board). Color: burnt sienna/tobacco. Year: 1871; d) bookbinding sample #55A is of unknown origin; a sample taken from the board: bottom left corner at tail center. Color: tan. Year: unknown. The black arrow indicates where samples were taken on the bookbinding samples.

Species identification in the bookbinding leather samples

Mass spectrometry data were analyzed using PEAKS Studio PEAKS XPro. Leather samples were searched against a compilated database of the *Triticum aestivum* proteome (proteome entry UP000019116, downloaded and compiled on July 13th, 2023, from Uniprot.org) to which were added the Uniprot proteomes of *Bos taurus* (UP000009136), *Ovis aries* (UP000002356), and *Capra hircus* (UP000291000) downloaded September 28th, 2023 from Uniprot.org. To the database were also added egg proteins from *Gallus gallus* (downloaded September 28th, 2023 from Uniprot.org) and collagen sequences for *Capra hircus* downloaded September 28th, 2023 from NCBI (https://www.ncbi.nlm.nih.gov/protein/). The parameters used were the following: 5 ppm parent mass error tolerance, 0.25 Da fragment mass error tolerance, 1 max missed cleavages, semispecific digest mode, fixed modifications: carbamidomethylation (C), variable modifications: oxidation (M), deamidation (N.Q.). Peptides were filtered using 1% FDR. Peaks PTM searches were performed to find further unspecified mutations. Three maximum variable PTMs were allowed per peptide. Proteins were further filtered with protein -10lgP ≥50.0 and a minimum of two peptides for protein validation.

A dedicated contaminant database was employed to identify and remove peptides originating from extraneous sources[1].

Tables S1 to S4 show peptides present in goat, sheep, and cattle, allowing differentiation between the three species. Only sequences with a minimum of three features and no missed cleavages are indicated. As per the manufacturer's description, markers of cattle collagen were found for sample V0 (Table S1), a cattle vegetable-tanned leather. **Sample 46 (Table S2)** is presumed to be made with goat leather based on the higher number of markers and features identified for *Capra* collagen. The identification of a *Capra* species is based on the identification of peptide GPSGEPGTAGPPGTPGPQGFLGPPGFLGLPGSR that has been used to differentiate goat from sheep[2]. The presence of cattle collagen is likely from hide glue, a common type of collagen glue[3]. **Sample 49 (Table S3)** can be identified as sheep leather based on the sequence GPSGEPGTAGPPGTPGPQGLLGAPGFLGLPGSR (in sheep but not goat, see above) and TGQPGAVGPAGIR (present in goat and sheep but not cattle). Finally, **sample 55** can be identified as cattle based on sequence IGQPGAVGPAGIR (in cattle but not sheep or goat), among other peptides (**Table S4**).

Table S1: Species markers in bookbinder sample V0. The markers present in cattle but not in sheep or goat are highlighted in blue.

Accession number	Protein	Peptide sequence	-10lgP score	Mass	Charge	ppm	# features	Position	Other species
sp|P02453|CO1A1_BOVIN	Collagen alpha-1(I) chain OS=Bos taurus OX=9913 GN=COL1A1 PE=1 SV=3	GEGGPQGPR	38.70	853.4042	2	-0.2	4	352-360	
		GEGGPQ(+.98)GPR	38.37	854.3882	2	0.3	3	352-360	
		GFPGADGVAGPK	49.85	1071.5349	2	0.2	4	493-504	
		GFP(+15.99)GADGVAGPK	44.47	1087.5298	2	0.8	4	493-504	
		GEAGPSGPAGPTGAR	68.58	1280.6108	2	-2.7	5	781-795	
		GPPGSAGSPGK	38.66	910.4508	2	0.4	3	1141-1151	
sp|P02465|CO1A2_BOVIN	Collagen alpha-2(I) chain OS=Bos taurus OX=9913 GN=COL1A2 PE=1 SV=2	GHAGLAGAR	40.35	808.4304	2	1.6	4	509-517	
		GAP(+15.99)GAIGAP(+15.99)GPAGANGDR	61.15	1536.7280	2	0.7	4	674-691	
		TGPP(+15.99)GPSGISGPP(+15.99)GPP(+15.99)GPAGK	65.79	1828.8955	2	0.3	7	793-813	
		TGP(+15.99)P(+15.99)GPSGISGPP(+15.99)GPP(+15.99)GPAGK	63.73	1844.8904	2	0.1	8	793-813	
		TGP(+15.99)PGP(+15.99)SGISGPP(+15.99)GPP(+15.99)GPAGK	44.06	1844.8904	2	0.1	3	793-813	
		SGETGASGPP(+15.99)GFVGEK	58.10	1491.6841	2	1.0	4	829-844	
		GPSGEP(+15.99)GTAGPP(+15.99)GTP(+15.99)GPQGLLGAP(+15.99)GFLGLP(+15.99)GSR	92.75	3032.4839	3	-3.2	3	845-877	Sheep
		GYP(+15.99)GNAGPVGAAGAP(+15.99)GPQGPVGPVGK	55.79	2260.1235	2	0.5	9	947-972	

		GYP(+15.99)GNAGPVGAAGAP(+15.99)GPQ(+.98)GPVGPVGK	54.70	2261.1077	3	0.3	4	947-972	
		GYP(+15.99)GN(+.98)AGPVGAAGAP(+15.99)GPQGPVGPVGK	49.41	2261.1077	2	1.2	6	947-972	
		GYPGN(+.98)AGP(+15.99)VGAAGAP(+15.99)GPQGPVGPVGK	43.95	2261.1077	2	0.2	3	947-972	
		GEP(+15.99)GPAGAVGPAGAVGPR	65.26	1531.7742	2	-0.1	5	977-994	
		GPAGPSGPAGK	49.88	894.4559	2	0.2	4	1052-1062	
		IGQPGAVGPAGIR	57.46	1191.6724	2	0.5	4	1066-1078	
		IGQ(+.98)PGAVGPAGIR	47.92	1192.6564	2	-0.4	4	1066-1078	
		IGQP(+15.99)GAVGPAGIR	50.84	1207.6672	2	0.7	4	1066-1078	
		IGQ(+.98)P(+15.99)GAVGPAGIR	43.11	1208.6512	2	0.4	3	1066-1078	
**tr|Q08E14|Q08E14_BOVIN**	**Collagen alpha-1(III) chain OS=Bos taurus OX=9913 GN=COL3A1 PE=2 SV=1**	GPP(+15.99)GPP(+15.99)GTNGVPGQR	50.25	1418.6902	2	0.5	4	414-428	
		GPP(+15.99)GPP(+15.99)GTNGVP(+15.99)GQR	50.40	1434.6851	2	0.2	4	414-428	
		GPP(+15.99)GPP(+15.99)GTNGVP(+15.99)GQ(+.98)R	44.92	1435.6691	2	0.7	4	414-428	
		GPP(+15.99)GPP(+15.99)GTN(+.98)GVP(+15.99)GQR	47.18	1435.6691	2	0.7	4	414-428	
		GEAGSP(+15.99)GIAGPK	42.81	1055.5247	2	0.3	3	450-461	Sheep
		DGASGHP(+15.99)GPIGPPGP(+15.99)R	58.48	1499.7117	3	1.2	3	1151-1166	

Table S2: Species markers in bookbinder sample 46A. The leather species is presumed to be goat based on the higher number of markers and features identified for goat collagen. The presence of cattle collagen is likely from hide glue. The markers present in goat but not sheep or cattle are highlighted in yellow, and those present in cattle but not in sheep or goat are highlighted in blue.

Accession number	Protein	Peptide sequence	-10lgP score	Mass	Charge	ppm	# features	Position	Other species
**XP_017920382.1**	**PREDICTED: collagen alpha-1(I) chain isoform X1 [Capra hircus]**	GEAGPQGPR	39.24	867.4199	2	0.6	5	352-360	Sheep
		GEAGPQ(+.98)GPR	34.51	868.4039	2	0.7	6	352-360	Sheep
		GFP(+15.99)GSDGVAGPK	52.74	1103.5247	2	0.4	6	493-504	Sheep
		GETGPSGPAGPTGAR	60.74	1310.6215	2	0.4	5	781-795	Sheep
		P(+15.99)GEVGPP(+15.99)GPP(+15.99)GPAGEK	39.34	1489.7048	2	1.0	5	918-933	Cattle
		GPP(+15.99)GSAGTPGK	43.14	940.4614	2	-0.1	6	1141-1151	Sheep
**sp|P02453|CO1A1_BOVIN**	**Collagen alpha-1(I) chain OS=Bos taurus OX=9913 GN=COL1A1 PE=1 SV=3**	GFP(+15.99)GADGVAGPK	46.54	1087.5298	2	0.1	10	493-504	
		GEAGPSGPAGPTGAR	56.33	1280.6108	2	0.8	4	781-795	
		P(+15.99)GEVGPP(+15.99)GPP(+15.99)GPAGEK	39.34	1489.7048	2	1.0	5	918-933	Goat
**XP_005678993.1**	**PREDICTED: collagen alpha-2(I) chain [Capra hircus]**	EGPAGLP(+15.99)GIDGR	35.93	1153.5728	2	0.7	5	463-474	Sheep
		GHAGLAGPR	49.49	834.4460	2	1.0	5	509-517	Sheep
		GAP(+15.99)GAVGAP(+15.99)GPAGAN(+.98)GDR	58.86	1523.6964	2	0.7	4	674-691	Sheep
		TGPP(+15.99)GPAGISGPP(+15.99)GPP(+15.99)GPAGK	59.87	1812.9005	2	-0.3	4	793-803	Sheep
		TGP(+15.99)P(+15.99)GPAGISGPP(+15.99)GPP(+15.99)GPAGK	68.13	1828.8955	2	-0.1	9	793-803	Sheep
		TGP(+15.99)P(+15.99)GP(+15.99)AGISGPP(+15.99)GPP(+15.99)GPAGK	41.13	1844.8904	2	0.7	3	793-803	Sheep
		TGP(+15.99)P(+15.99)GPAGISGP(+15.99)P(+15.99)GPP(+15.99)GPAGK	37.65	1844.8904	2	-0.1	3	793-803	Sheep
		TGEP(+15.99)GAAGPP(+15.99)GFVGEK	72.92	1501.7048	2	0.1	5	829-844	Sheep
		GPSGEP(+15.99)GTAGPP(+15.99)GTP(+15.99)GPQGFLGPP(+15.99)GFLGLP(+15.99)GSR	63.86	3092.4839	3	0.7	4	845-877	
		GPSGEP(+15.99)GTAGPP(+15.99)GTPGP(+15.99)Q(+.98)GFLGPP(+15.99)GFLGLP(+15.99)GSR	52.68	3093.4678	3	0.4	3	845-877	
		GPSGEP(+15.99)GTAGP(+15.99)PGTP(+15.99)GPQ(+.98)GFLGPP(+15.99)GFLGLP(+15.99)GSR	57.66	3093.4678	3	0.4	4	845-877	
		GYP(+15.99)GN(+.98)AGPVGAAGAP(+15.99)GPQGPVGPTGK	38.79	2263.0869	2	-0.2	7	947-972	Sheep
		GYPGN(+.98)AGP(+15.99)VGAAGAP(+15.99)GPQGPVGPTGK	31.14	2263.0869	3	0.1	4	947-972	Sheep
		GYP(+15.99)GN(+.98)AGPVGAAGAP(+15.99)GPQ(+.98)GPVGPTGK	44.79	2264.0708	2	0.3	7	947-972	Sheep
		GEP(+15.99)GPVGAVGPAGAVGPR	66.96	1559.8055	2	-0.2	5	977-994	Sheep
		GPAGPTGPAGK	52.81	908.4716	2	1.2	5	1052-1062	Sheep
		G(+57.02)PAGPTGPAGK	37.03	965.4930	2	1.0	3	1052-1062	Sheep
		TGQPGAVGPAGIR	68.51	1179.6360	2	0.4	5	1066-1078	Sheep
		TGQ(+.98)PGAVGPAGIR	53.56	1180.6200	2	-0.1	5	1066-1078	Sheep
		TGQP(+15.99)GAVGPAGIR	50.13	1195.6309	2	1.1	5	1066-1078	Sheep
		TGQ(+.98)P(+15.99)GAVGPAGIR	54.42	1196.6149	2	0.7	4	1066-1078	Sheep
**sp|P02465|CO1A2_BOVIN**	**Collagen alpha-2(I) chain OS=Bos taurus OX=9913 GN=COL1A2 PE=1 SV=2**	GHAGLAGAR	38.25	808.4304	2	-0.1	4	509-517	
		TGPPGPSGISGPP(+15.99)GPP(+15.99)GPAGK	38.60	1812.9005	2	1.3	4	793-813	
		TGP(+15.99)PGPSGISGPP(+15.99)GPP(+15.99)GPAGK	52.46	1828.8955	2	0.0	3	793-813	
		TGP(+15.99)P(+15.99)GPSGISGPP(+15.99)GPP(+15.99)GPAGK	57.67	1844.8904	2	0.3	6	793-813	
		SGETGASGPP(+15.99)GFVGEK	58.53	1491.6841	2	2.0	4	829-844	
		GPSGEP(+15.99)GTAGPP(+15.99)GTP(+15.99)GPQ(+.98)GLLGAP(+15.99)GFLGLP(+15.99)GSR	64.34	3033.4678	3	0.4	3	845-877	Sheep
		GYP(+15.99)GN(+.98)AGPVGAAGAP(+15.99)GPQGPVGPVGK	48.29	2261.1077	2	0.6	5	947-972	
		GYP(+15.99)GN(+.98)AGPVGAAGAP(+15.99)GPQ(+.98)GPVGPVGK	43.77	2262.0916	2	0.3	7	947-972	
		GEP(+15.99)GPAGAVGPAGAVGPR	53.15	1531.7742	2	-0.1	3	977-994	
		GPAGPSGPAGK	47.44	894.4559	2	0.3	4	1052-1062	
		IGQPGAVGPAGIR	57.05	1191.6724	2	0.1	5	1066-1078	
		IGQ(+.98)PGAVGPAGIR	51.46	1192.6554	2	0.0	5	1066-1078	
		IGQP(+15.99)GAVGPAGIR	50.48	1207.6672	2	0.3	5	1066-1078	
		IGQ(+.98)P(+15.99)GAVGPAGIR	45.87	1208.6512	2	0.2	5	1066-1078	
**XP_005675926.1**	**PREDICTED: collagen alpha-1(III) chain [Capra hircus]**	GPP(+15.99)GPP(+15.99)GTN(+.98)GAP(+15.99)GQR	49.91	1407.6378	2	0.6	4	415-429	Sheep
		GPP(+15.99)GPP(+15.99)GTN(+.98)GAP(+15.99)GQ(+.98)R	40.78	1408.6218	2	0.4	5	415-429	Sheep
		GEAGSP(+15.99)GIP(+15.99)GPK	48.29	1097.5353	2	-0.1	5	451-462	
		GEGGAP(+15.99)GVP(+15.99)GIAGPR	48.55	1322.6578	2	0.5	3	781-795	Sheep
		DGTSGHPGPIGPP(+15.99)GPR	59.99	1513.7273	3	0.8	6	1152-1167	Sheep
		DGTSGHP(+15.99)GPIGPP(+15.99)GPR	42.41	1529.7222	3	1.0	6	1152-1167	Sheep
**tr|Q08E14|Q08E14_BOVIN**	**Collagen alpha-1(III) chain OS=Bos taurus OX=9913 GN=COL3A1 PE=2 SV=1**	GPP(+15.99)GPP(+15.99)GTN(+.98)GVP(+15.99)GQ(+.98)R	43.77	1436.6532	2	0.3	3	414-428	
		GEAGSP(+15.99)GIAGPK	40.78	1055.5247	2	1.2	4	450-461	Sheep
		GPP(+15.99)GPQGPR	23.61	877.4406	2	0.5	3	1083-1091	
		DGASGHPGPIGPP(+15.99)GPR	63.90	1483.7167	3	1.0	3	1151-1166	
		DGASGHP(+15.99)GPIGPP(+15.99)GPR	63.34	1499.7117	3	0.4	5	1151-1166	

Table S3: Species markers in bookbinder sample 49A. The leather species is presumed to be sheep based on the higher number of markers and features identified for Sheep collagen. The markers present in sheep but not in goat or cattle are highlighted in green.

Accession number	Protein	Peptide sequence	-10lgP score	Mass	Charge	ppm	# features	Position	Other species
tr|W5P481|W5P481_SHEEP	Uncharacterized protein OS=Ovis aries OX=9940 GN=COL1A1 PE=4 SV=1	GEAGPQ(+.98)GPR	29.30	868.4039	2	0.4	4	360-368	Goat
		GFP(+15.99)GSDGVAGPK	48.88	1103.5247	2	0.4	6	501-512	Goat
		GETGPSGPAGPTGAR	58.31	1310.6215	2	0.8	4	789-803	Goat
		AGEVGPP(+15.99)GPPGPAGEK	48.94	1431.6993	2	0.9	4	926-941	
		AGEVGPP(+15.99)GPP(+15.99)GPAGEK	43.75	1447.6942	2	0.3	7	926-941	
		AGEVGPP(+15.99)GP(+15.99)P(+15.99)GPAGEK	37.70	1463.6892	2	0.8	4	926-941	
		AGEVGPP(+15.99)GPP(+15.99)GP(+15.99)AGEK	39.59	1463.6892	2	0.4	5	926-941	
		AGEVGPP(+15.99)GP(+15.99)PGP(+15.99)AGEK	36.02	1463.6892	2	0.8	3	926-941	
		GPPGSAGTPGK	39.26	924.4664	2	0.1	3	1148-1158	Goat
		GPP(+15.99)GSAGTPGK	44.28	940.4614	2	0.2	3	1148-1158	Goat
tr|W5NTT7|W5NTT7_SHEEP	Fibrillar collagen NC1 domain-containing protein OS=Ovis aries OX=9940 GN=COL1A2 PE=4 SV=1	EGPAGLP(+15.99)GIDGR	37.47	1153.5728	2	0.7	4	463-474	Goat
		GHAGLAGPR	43.12	834.4460	2	1.0	5	509-517	Goat
		GAP(+15.99)GAVGAP(+15.99)GPAGANGDR	56.73	1522.7124	2	1.1	3	674-691	Goat
		GAP(+15.99)GAVGAP(+15.99)GPAGAN(+.98)GDR	60.81	1523.6964	2	0.3	3	674-691	Goat
		TGPP(+15.99)GPAGISGPP(+15.99)GPP(+15.99)GPAGK	62.09	1812.9005	2	0.7	5	793-813	Goat
		TGP(+15.99)P(+15.99)GPAGISGPP(+15.99)GPP(+15.99)GPAGK	62.46	1828.8955	2	1.0	12	793-813	Goat
		TGEP(+15.99)GAAGPP(+15.99)GFVGEK	67.30	1501.7048	2	0.7	6	829-844	Goat
		GPSGEP(+15.99)GTAGPP(+15.99)GTPGPQ(+.98)GLLGAP(+15.99)GFLGLP(+15.99)GSR	64.05	3017.4729	3	0.6	3	845-877	Cattle
		GPSGEP(+15.99)GTAGP(+15.99)PGTP(+15.99)GPQGLLGAP(+15.99)GFLGLP(+15.99)GSR	64.87	3032.4839	3	1.2	4	845-877	Cattle
		GPSGEP(+15.99)GTAGPP(+15.99)GTP(+15.99)GPQGLLGAP(+15.99)GFLGLP(+15.99)GSR	73.82	3032.4839	3	0.7	5	845-877	Cattle
		GPSGEP(+15.99)GTAGPP(+15.99)GTP(+15.99)GPQ(+.98)GLLGAP(+15.99)GFLGLP(+15.99)GSR	74.06	3033.4678	3	-0.1	5	845-877	Cattle
		GPSGEP(+15.99)GTAGP(+15.99)PGTP(+15.99)GPQ(+.98)GLLGAP(+15.99)GFLGLP(+15.99)GSR	60.59	3033.4678	3	1.2	3	845-877	Cattle
		GYP(+15.99)GNAGPVGAAGAP(+15.99)GPQ(+.98)GPVGPTGK	55.01	2263.0869	3	0.7	7	947-972	Goat
		GYPGNAGP(+15.99)VGAAGAP(+15.99)GPQ(+.98)GPVGPTGK	50.31	2263.0869	3	1.0	3	947-972	Goat
		GYP(+15.99)GN(+.98)AGPVGAAGAP(+15.99)GPQGPVGPTGK	46.63	2263.0869	2	0.5	8	947-972	Goat
		GYP(+15.99)GN(+.98)AGPVGAAGAP(+15.99)GPQ(+.98)GPVGPTGK	57.07	2264.0708	3	0.6	10	947-972	Goat
		GYPGN(+.98)AGP(+15.99)VGAAGAP(+15.99)GPQ(+.98)GPVGPTGK	31.65	2264.0708	2	1.8	3	947-972	Goat
		GEP(+15.99)GPVGAVGPAGAVGPR	64.95	1559.8055	2	0.0	6	977-994	Goat
		GEP(+15.99)GP(+15.99)VGAVGPAGAVGPR	48.20	1575.8004	2	0.8	7	977-994	Goat
		GEP(+15.99)GPVGAVGPAGAVGP(+15.99)R	53.90	1575.8004	2	0.8	4	977-994	Goat
		GEP(+15.99)GPVGAVGP(+15.99)AGAVGPR	46.85	1575.8004	2	0.8	3	977-994	Goat
		GPAGPTGPAGK	48.74	908.4716	2	1.2	5	1052-1062	Goat
		G(+57.02)PAGPTGPAGK	36.14	965.4930	2	1.0	3	1052-1062	Goat
		TGQPGAVGPAGIR	60.03	1179.6360	2	0.0	3	1066-1078	Goat
		TGQ(+.98)PGAVGPAGIR	47.97	1180.6200	2	1.0	5	1066-1078	Goat
		TGQP(+15.99)GAVGPAGIR	45.43	1195.6309	2	0.6	4	1066-1078	Goat
		TGQ(+.98)P(+15.99)GAVGPAGIR	50.61	1196.6149	2	1.1	5	1066-1078	Goat
tr|W5Q4S0|W5Q4S0_SHEEP 1	Uncharacterized protein OS=Ovis aries OX=9940 GN=COL3A1 PE=4 SV=	GPP(+15.99)GPP(+15.99)GTNGAP(+15.99)GQ(+.98)R	48.23	1407.6378	2	0.2	3	415-429	Goat
		GPP(+15.99)GPP(+15.99)GTN(+.98)GAP(+15.99)GQ(+.98)R	41.50	1408.6218	2	1.0	5	415-429	Goat
		GEAGSP(+15.99)GIAGPK	43.63	1055.5247	2	1.2	4	451-462	Cattle
		GEP(+15.99)GPP(+15.99)GPAGFP(+15.99)GAP(+15.99)GQ(+.98)NGEP(+15.99)GAK	39.10	2222.9714	3	1.5	5	802-825	Goat
		GPP(+15.99)GPQ(+.98)GLP(+15.99)GLAGAAGEP(+15.99)GR	56.14	1803.8750	2	0.5	3	994-1013	Goat
		DGTSGHPGPIGPP(+15.99)GPR	59.50	1513.7273	3	1.5	8	1152-1167	Goat
		DGTSGHP(+15.99)GPIGPP(+15.99)GPR	50.85	1529.7222	3	0.4	8	1152-1167	Goat

Table S4: Species markers in bookbinder sample 55A. The leather species is presumed to be cattle based on the higher number of markers and features identified for cattle collagen. The markers present in cattle but not in sheep or goat are highlighted in blue.

Accession number	Protein	Peptide sequence	-10lgP score	Mass	Charge	ppm	# features	Position	Other species
sp|P02453|CO1A1_BOVIN	Collagen alpha-1(I) chain OS=Bos taurus OX=9913 GN=COL1A1 PE=1 SV=3	GFPGADGVAGPK	48.53	1071.5349	2	1.1	4	493-504	
		GFP(+15.99)GADGVAGPK	47.53	1087.5298	2	1.1	6	493-504	
		GFP(+15.99)GADGVAGPK(+42.02)	29.11	1129.5515	2	0.5	4	493-504	
		GEAGPSGPAGPTGAR	68.23	1280.6108	2	0.6	5	781-795	
		P(+15.99)GEVGPP(+15.99)GPP(+15.99)GPAGEK	41.47	1489.7048	2	-0.3	4	918-933	Goat
sp|P02465|CO1A2_BOVIN	Collagen alpha-2(I) chain OS=Bos taurus OX=9913 GN=COL1A2 PE=1 SV=2	EGPVGLP(+15.99)GIDGR	38.69	1181.6040	2	0.8	5	463-474	
		GHAGLAGAR	38.87	808.4304	2	0.0	4	509-517	
		GAP(+15.99)GAIGAP(+15.99)GPAGANGDR	53.17	1536.7280	2	0.8	5	674-691	
		GAP(+15.99)GAIGAP(+15.99)GPAGAN(+.98)GDR	60.46	1537.7120	2	0.0	7	674-691	
		TGPP(+15.99)GPSGISGPP(+15.99)GPP(+15.99)GPAGK	65.19	1828.8955	2	0.7	5	793-813	
		TGP(+15.99)PGP(+15.99)SGISGPP(+15.99)GPP(+15.99)GPAGK	37.52	1844.8904	3	0.3	4	793-813	
		TGP(+15.99)P(+15.99)GPSGISGPP(+15.99)GPP(+15.99)GPAGK	61.55	1844.8904	2	0.1	9	793-813	
		SGETGASGPP(+15.99)GFVGEK	62.29	1491.6841	2	0.1	5	829-844	
		GPSGEP(+15.99)GTAGPP(+15.99)GTP(+15.99)GPQGLLGAP(+15.99)GFLGLP(+15.99)GSR	67.58	3032.4839	3	0.4	5	845-877	Sheep
		GPSGEP(+15.99)GTAGP(+15.99)PGTP(+15.99)GPQ(+.98)GLLGAP(+15.99)GFLGLP(+15.99)GSR	57.34	3033.4678	3	0.0	3	845-877	Sheep
		GPSGEP(+15.99)GTAGPP(+15.99)GTP(+15.99)GPQ(+.98)GLLGAP(+15.99)GFLGLP(+15.99)GSR	63.32	3033.4678	3	0.6	3	845-877	Sheep
		GYPGNAGP(+15.99)VGAAGAP(+15.99)GPQGPVGPVGK	42.63	2260.1235	3	0.1	3	947-972	
		GYP(+15.99)GNAGPVGAAGAP(+15.99)GPQGPVGPVGK	58.48	2260.1235	2	-0.2	6	947-972	
		GYP(+15.99)GNAGPVGAAGAP(+15.99)GPQ(+.98)GPVGPVGK	52.66	2261.1077	3	0.7	8	947-972	
		GYP(+15.99)GN(+.98)AGPVGAAGAP(+15.99)GPQGPVGPVGK	60.22	2261.1077	3	0.2	7	947-972	
		GYP(+15.99)GN(+.98)AGPVGAAGAP(+15.99)GPQ(+.98)GPVGPVGK	63.87	2262.0916	3	1.0	10	947-972	
		GEP(+15.99)GPAGAVGPAGAVGPR	64.89	1531.7742	2	-0.1	6	977-994	
		GPAGPSGPAGK	44.87	894.4559	2	0.7	5	1052-1062	
		IGQPGAVGPAGIR	59.03	1191.6724	2	1.0	5	1066-1078	
		IGQ(+.98)PGAVGPAGIR	53.64	1192.6564	2	0.2	5	1066-1078	
		IGQP(+15.99)GAVGPAGIR	53.05	1207.6672	2	0.4	6	1066-1078	
		IGQ(+.98)P(+15.99)GAVGPAGIR	53.57	1208.6512	2	0.6	7	1066-1078	
tr|Q08E14|Q08E14_BOVIN	Collagen alpha-1(III) chain OS=Bos taurus OX=9913 GN=COL3A1 PE=2 SV=1	GPP(+15.99)GPP(+15.99)GTN(+.98)GVPGQ(+.98)R	36.47	1420.6582	2	0.2	4	414-428	
		GPP(+15.99)GPP(+15.99)GTNGVP(+15.99)GQ(+.98)R	50.31	1435.6691	2	0.4	5	414-428	
		GPP(+15.99)GPP(+15.99)GTN(+.98)GVP(+15.99)GQR	52.81	1435.6691	2	0.7	6	414-428	
		GPP(+15.99)GPP(+15.99)GTN(+.98)GVP(+15.99)GQ(+.98)R	43.95	1436.6532	2	0.5	5	414-428	
		GEAGSP(+15.99)GIAGPK	42.86	1055.5247	2	-0.3	5	450-461	Sheep
		GESGAP(+15.99)GVP(+15.99)GIAGPR	40.62	1352.6683	2	0.9	3	780-794	
		GFPGN(+.98)P(+15.99)GAP(+15.99)GSP(+15.99)GPAGHQGAVGSP(+15.99)GPAGPR	28.52	2669.2217	3	-0.2	3	1110-1139	Goat
		GFP(+15.99)GNPGAP(+15.99)GSP(+15.99)GPAGHQ(+.98)GAVGSP(+15.99)GPAGPR	45.04	2669.2217	3	-0.2	3	1110-1139	Goat
		GFPGN(+.98)P(+15.99)GAP(+15.99)GSP(+15.99)GPAGHQ(+.98)GAVGSP(+15.99)GPAGPR	39.21	2670.2058	3	0.5	4	1110-1139	Goat
		GFP(+15.99)GN(+.98)PGAP(+15.99)GSP(+15.99)GPAGHQ(+.98)GAVGSP(+15.99)GPAGPR	48.64	2670.2058	3	0.5	3	1110-1139	Goat
		GFP(+15.99)GNP(+15.99)GAP(+15.99)GSP(+15.99)GPAGHQGAVGSP(+15.99)GPAGPR	51.65	2684.2327	3	0.4	3	1110-1139	Goat
		GFP(+15.99)GNP(+15.99)GAP(+15.99)GSP(+15.99)GPAGHQ(+.98)GAVGSP(+15.99)GPAGPR	47.72	2685.2166	3	0.3	4	1110-1139	Goat
		DGASGHPGPIGPP(+15.99)GPR	70.27	1483.7167	3	0.6	6	1151-1166	
		DGASGHP(+15.99)GPIGPPGP(+15.99)R	52.91	1499.7117	3	-0.2	3	1151-1166	
		DGASGHP(+15.99)GPIGPP(+15.99)GPR	60.35	1499.7117	3	1.3	8	1151-1166	

Identification of milk proteins

Milk proteins, i.e., casein and lactoglobulin, were identified in all samples in trace amounts or as an apparent additive to the materials tested.

To address potential concerns regarding laboratory contamination, we implemented several stringent measures throughout our sample processing and analysis protocols:

Protocol blanks: We processed protocol blanks alongside our samples to monitor for contamination during sample preparation, extraction, and analysis phases. Peptides originating from extraneous sources were incorporated into the dedicated contaminant database.

Reagents: All reagents used were of analytical grade, and batches were tracked to identify any potential familiar sources of contamination. Reagents known to carry proteinaceous contaminants (e.g., trypsin) were potentially sourced from suppliers that guarantee low endogenous contamination levels.

A- Milk in starch

Milk was found in combined samples of starch glue. Certain commercial flour-derived products come with the warning "May contain: Milk" due to processing facilities where milk is present. The starch used in this study did not contain information regarding potential traces of milk. However, four milk proteins were identified in starch glue (**Table S5a**) and many peptides (**Table S5b**). This abundance of peptides indicates this is not laboratory contamination. However, it could be more substantial contamination from the manufacturer, and its presence could be enhanced in our analysis due to the relatively low amount of proteins in the starch product.

Table S5a: Protein identification in Starch glue

Protein Group	Accession	-10lgP	% Cov	#Pept	#Spec	Description
20	P02662	196.17	43	10	30	Alpha-S1-casein OS=Bos taurus OX=9913 GN=CSN1S1 PE=1 SV=2
43	tr|A0A452DHW7	107.87	29	5	10	Beta-casein OS=Bos taurus OX=9913 GN=CSN2 PE=1 SV=1
57	P02754	102.93	34	3	7	Beta-lactoglobulin OS=Bos taurus OX=9913 GN=LGB PE=1 SV=3
84	P02663	51.09	9	2	3	Alpha-S2-casein OS=Bos taurus OX=9913 GN=CSN1S2 PE=1 SV=2

Table S5b: Peptide identification in Starch glue

Protein Group	Peptide	-10lgP	Mass	# Feature	Start	End	Blast genera
20	K.H(+57.02)QGLPQEVLNENLLR.F	76.53	1815.959	1	23	37	*Bos; Bison; Muntiacus; Jeotgalicoccus*
	R.F(+57.02)FVAPFPEVFGK.E	64.76	1440.744	1	38	49	*Bos; Bison; Bubalus; Jeotgalicoccus*
	R.YLGY(+125.90)LEQLLR.L	29.87	1392.594	1	106	115	*Bos; Bison; Bubalus; Jeotgalicoccus; Capra; Ovis; Budorcas; Moschus*
	K.YKVPQLEIVPNS(+79.97)AEER.L	65.28	1950.945	1	119	134	*Bos; Bison; Jeotgalicoccus*
	K.YK(+57.02)VPQLEIVPNS(+79.97)AEER.L	49.45	2007.967	1	119	134	
	K.VPQLEIVPNS(+79.97)AEER.L	49.21	1659.787	1	121	134	
	K.EPM(+15.99)IGVNQELAYFYPELFR.Q	60.05	2331.125	2	148	166	*Bos; Bison; Jeotgalicoccus*
	K.EPMIGVNQE(+57.02)LAYFYPELFR.Q	36.47	2372.151	0	148	166	
	K.EP(+15.99)MIGVNQELAYFYPELFR.Q	35.12	2331.125	0	148	166	
	M.IGVNQELAYFYPELFR.Q	39.05	1957.994	1	151	166	
	N.QELAYFYPELFR.Q	31.64	1574.777	0	155	166	
	Y.TDAPSFSDIPNPIGSENSEK.T	49.09	2103.96	2	189	208	*Bos; Jeotgalicoccus*
	D.APSFSDIPNPIGSENSEK.T	43.74	1887.885	1	191	208	
43	R.D(+43.01)M(+15.99)PIQAFLLYQEPVLGPVR.G	56.2	2244.161	1	234	252	*Bos; Bison; Bubalus; Capra; Ovis; Budorcas; Oryx*
	R.D(+57.02)MPIQAFLLYQEPVLGPVR.G	47.36	2242.182	2	234	252	
	M.P(+57.02)IQAFLLYQEPVLGPVR.G	48.19	1996.115	0	236	252	
	F.LLYQEPVLGPVR.G	40.94	1382.792	1	241	252	
	F.L(+57.02)LYQEPVLGPVR.G	39.45	1439.814	1	241	252	
	F.LLY(+125.90)QEPVLGPVR.G	30.25	1508.689	1	241	252	
	G.PVRGPFPIIV	33.64	1093.665	1	250	259	*Bos; Bison; Bubalus; Rangifer; Oryx; Cervus*
57	K.VAGTWYSLAM(+15.99)AASDISLLDAQSAPLR.V	59.9	2722.364	1	31	56	*Bos; Bison; Bubalus;*

	K.VAGTWYSLAMAASD(+57.02)ISLLDAQSAPLR.V	45.49	2763.39	1	31	56	*Staphylococcus; Capra; Ovis; Rangifer; Budorcas; Moschus; Muntiacus; Dama; Oryx; Cervus*
	R.VYVEELKPTPEGDLEILLQK.W	35.74	2312.252	2	57	76	*Bos; Bison; Bubalus; Staphylococcus; Capra; Ovis; Moschus; Muntiacus; Oryx*
	R.TPEVDDEALEKFDK.A	61.06	1634.768	1	141	154	*Bos; Bison; Bubalus; Staphylococcus; Jeotgalicoccus*
84	K.ALNEINQFYQK.F	34.48	1366.688	1	96	106	*Bos; Bison; Bubalus; Jeotgalicoccus; Capra; Ovis; Budorcas*
	K.FALPQYLK.T	33.22	978.5538	1	189	196	*Bos; Bison; Jeotgalicoccus ; Enterobacter; Leclercia; Escherichia; Trabulsiella; Marinobacter; Candidatus*

B- Milk in Leather samples

Milk was found in all leather samples at levels above trace contamination. Indeed, in all samples, except for 46A, more than one milk protein was identified. All samples were consistent with cattle milk, based on the Blast analysis against all organisms (with https://blast.ncbi.nlm.nih.gov/Blast.cgi). Cross-contamination between samples is unlikely due to the identification of different sets of proteins and peptides in each sample. Blanks were run before and after each leather sample that did not contain casein. Cross-contamination with starch glue is also unlikely, as samples were prepared separately and analyzed at different times.

Smaller sub-samples (1 x 1 mm) of the same bookbinding leathers were independently analyzed in a separate laboratory (Smithsonian Conservation Institute) equipped with a different mass spectrometer and using different reagents (unpublished data). Milk peptides were found in samples 46A and 49A but not 55A (V0 was not tested). The replication of this finding in at least two leather samples in an entirely separate laboratory setting mitigates the possibility of our results being contamination artifacts specific to our laboratory or analytical equipment. This cross-validation strongly suggests that the presence of milk proteins in the samples is a genuine occurrence, not influenced by local laboratory conditions or methodologies. The absence of milk in 55A in the previous analysis might be due to the small sample size and variations in the specific location of these proteins.

Table S6a. Protein identification in V0

Protein Group	Accession	-10lgP	% Cov	#Pept	#Spec	Description
68	P02668	67.95	15	2	6	Kappa-casein OS=Bos taurus OX=9913 GN=CSN3 PE=1 SV=1
74	P02754	73.99	22	2	5	Beta-lactoglobulin OS=Bos taurus OX=9913 GN=LGB PE=1 SV=3

Table S6b: Peptide identification in V0

Protein Group	Peptide	-10lgP	Mass	# Feature	Start	End	Blast genera
68	K.YIPIQYVLSR.Y	47.98	1250.702	2	46	55	*Non-specific*
	R.SPAQILQWQVLSNTVPAK.S	39.94	1979.084	1	90	107	*Bos; Bison; Boselaphus ; Tetracerus ; Bubalus*
74	K.VAGTWYSLAM(+15.99)AASDISLLDAQSAPLR.V	57.03	2722.364	1	31	56	*Bos; Bison; Bubalus; Staphylococcus; Capra; Ovis; Rangifer; Budorcas; Moschus; Muntiacus; Dama; Oryx; Cervus*
	R.LSFNPTQLEEQC(+57.02)HI	33.9	1714.799	2	165	178	*Bos; Bubalus; Staphylococcus; Jeotgalicoccus; Moschus*

Table S7a: Protein identification in 46A

Protein Group	Accession	-10lgP	% Cov	#Pept	#Spec	Description
27	P02662	115.27	19	4	30	Alpha-S1-casein OS=Bos taurus OX=9913 GN=CSN1S1 PE=1 SV=2

Table S7b: Peptide identification in 46A

Protein Group	Peptide	-10lgP	Mass	# Feature	Start	End	Species

27	R.YLGYLEQ(+.98)LLR.L	52.77	1267.681	3	106	115	*Bos; Bison; Bubalus; Jeotgalicoccus; Capra; Ovis; Budorcas; Moschus*
	K.EPM(+15.99)IGVNQELAYFYPELFR.Q	27.85	2331.125	2	148	166	*Bos; Bison; Jeotgalicoccus*
	K.EP(+15.99)MIGVNQ(+.98)ELAYFYPELFR.Q	21.31	2332.108	0	148	166	
	M.IGVNQELAYFYPELFR.Q	21.47	1957.994	0	151	166	

Table S8a: Protein identification in 49A

Protein Group	Accession	-10lgP	% Cov	#Pept	#Spec	Description
64	P02662	135.11	30	5	40	Alpha-S1-casein OS=Bos taurus OX=9913 GN=CSN1S1 PE=1 SV=2
169	P02754	73.55	34	3	4	Beta-lactoglobulin OS=Bos taurus OX=9913 GN=LGB PE=1 SV=3

Table S8b: Peptide identification in 49A

Protein Group	Peptide	-10lgP	Mass	# Feature	Start	End	Species
64	R.YLGYLEQ(+.98)LLR.L	46.71	1267.681	3	106	115	*Bos; Bison; Bubalus; Jeotgalicoccus; Capra; Ovis; Budorcas; Moschus*
	K.EGIHAQQKEPMIGVNQELAYFYPELFR.Q	58.81	3206.586	3	140	166	*Bos; Bison; Jeotgalicoccus*
	K.EGIHAQQKEPM(+15.99)IGVNQELAYFYPELFR.Q	30.23	3222.581	1	140	166	
	K.EPMIGVNQELAYFYPELFR.Q	48.53	2315.13	4	148	166	
	K.EPMIGVN(+.98)QELAYFYPELFR.Q	38.67	2316.114	0	148	166	
169	K.VAGTWYSLAMAASDISLLDAQSAPLR.V	31.14	2706.369	1	31	56	*Bos; Bison; Bubalus; Staphylococcus; Capra; Ovis; Rangifer; Budorcas; Moschus; Muntiacus; Dama; Oryx; Cervus*
	R.VYVEELKPTPEGDLEILLQK.W	48.72	2312.252	1	57	76	*Bos; Bison; Bubalus; Staphylococcus; Capra; Ovis; Moschus; Muntiacus; Oryx*
	R.TPEVDDEALEKFDK.A	27.77	1634.768	1	141	154	*Bos; Bison; Bubalus; Staphylococcus; Jeotgalicoccus*

Table S9a: Protein identification in 55A

Protein Group	Accession	-10lgP	% Cov	#Pept	#Spec	Description
44	P02662	147.06	37	8	40	Alpha-S1-casein OS=Bos taurus OX=9913 GN=CSN1S1 PE=1 SV=2
64	P02666	82.25	17	2	21	Beta-casein OS=Bos taurus OX=9913 GN=CSN2 PE=1 SV=2
93	P02754	86.21	26	2	11	Beta-lactoglobulin OS=Bos taurus OX=9913 GN=LGB PE=1 SV=3

Table S9b: Peptide identification in 55A

Protein Group	Peptide	-10lgP	Mass	# Feature	Start	End	Species
44	R.F(+57.02)FVAPFPEVFGK.E	45.03	1440.744	1	38	49	*Bos; Bison; Bubalus; Jeotgalicoccus*
	R.FFVAPFPEVFGKEK.V	42.7	1640.86	1	38	51	
	K.EGIHAQQKEPMIGVNQELAYFYPELFR.Q	57.6	3206.586	1	140	166	*Bos; Bison; Jeotgalicoccus*

	K.EGIHAQQKEPMIGVNQ(+.98)ELAYFYPELFR.Q	47.48	3207.57	0	140	166	
	K.EGIHAQQKEP(+15.99)MIGVNQELAYFYPELFR.Q	42.03	3222.581	1	140	166	
	K.EPMIGVNQELAYFYPELFR.Q	55.47	2315.13	2	148	166	
	K.EPM(+15.99)IGVNQELAYFYPELFR.Q	38.91	2331.125	1	148	166	
	M.IGVNQELAYFYPELFR.Q	31.22	1957.994	1	151	166	
	W.YYVPLGTQYTDAPSFSDIPNPIGSENSEK.T	25.27	3188.482	1	180	208	*Bos; Jeotgalicoccus*
	Y.YVPLGTQYTDAPSFSDIPNPIGSENSEK.T	30.16	3025.419	1	181	208	
64	K.IHPFAQTQSLVYPFPGPIPN.S	27.51	2222.152	1	64	83	*Bos; Bison; Bubalus*
	R.DM(+15.99)PIQAFLLYQEPVLGPVR.G	68.49	2201.156	3	199	217	*Bos; Bison; Bubalus; Capra; Ovis; Oryx*
	R.D(+57.02)M(+15.99)PIQAFLLYQEPVLGPVR.G	58.28	2258.177	2	199	217	
	R.DMPIQAFLLYQEPVLGPVR.G	55.23	2185.16	3	199	217	
	R.D(+21.98)M(+15.99)PIQAFLLYQEPVLGPVR.G	21.86	2223.138	1	199	217	
	R.D(+57.02)MPIQAFLLYQEPVLGPVR.G	21.68	2242.182	1	199	217	
93	K.VAGTWYSLAMAASDISLLDAQSAPLR.V	47.63	2706.369	0	31	56	*Bos; Bison; Bubalus; Staphylococcus; Capra; Ovis; Rangifer; Budorcas; Moschus; Muntiacus; Dama; Oryx; Cervus*
	K.VAGTWYSLAM(+15.99)AASDISLLDAQSAPLR.V	61.35	2722.364	2	31	56	
	R.VYVEELKPTPEGDLEILLQK.W	49.73	2312.252	1	57	76	*Bos; Bison; Bubalus; Staphylococcus; Capra; Ovis; Moschus; Muntiacus; Oryx*

References

(1) Frankenfield, A. M.; Ni, J.; Ahmed, M.; Hao, L. Protein Contaminants Matter: Building Universal Protein Contaminant Libraries for DDA and DIA Proteomics. *J. Proteome Res.* **2022**, *21* (9), 2104–2113. DOI:/10.1021/acs.jproteome.2c00145.

(2) Buckley, M.; Whitcher Kansa, S.; Howard, S.; Campbell, S.; Thomas-Oates, J.; Collins, M. Distinguishing between Archaeological Sheep and Goat Bones Using a Single Collagen Peptide. *J. Archaeol. Sci.* **2010**, *37* (1), 13–20. DOI:10.1016/j.jas.2009.08.020.

(3) Ntasi, G.; Sbriglia, S.; Pitocchi, R.; Vinciguerra, R.; Melchiorre, C.; Dello Ioio, L.; Fatigati, G.; Crisci, E.; Bonaduce, I.; Carpentieri, A.; Marino, G.; Birolo, L. Proteomic Characterization of Collagen-Based Animal Glues for Restoration. *J. Proteome Res.* **2022**, *21* (9), 2173–2184. DOI:10.1021/acs.jproteome.2c00232.
